# Effect of antimicrobial growth promoter administration on the intestinal microbiota of beef cattle

**DOI:** 10.1186/1757-4749-5-8

**Published:** 2013-04-11

**Authors:** Kristen L Reti, Matthew C Thomas, L Jay Yanke, L Brent Selinger, G Douglas Inglis

**Affiliations:** 1 Agriculture and Agri-Food Canada Research Centre, 5403-1st Avenue S, Lethbridge, AB, T1J 4B1, Canada; 2 Department of Biological Sciences, University of Lethbridge, 4401 University Drive West, Lethbridge, AB, T1K 3M4, Canada

**Keywords:** Antimicrobial growth promoters, AGP, Beef cattle, Intestine, Microbiota

## Abstract

**Background:**

Antimicrobial growth promoters (AGPs) are antimicrobial agents administered to livestock in feed for prolonged periods to enhance feed efficiency. Beef cattle are primarily finished in confined feeding operations in Canada and the USA, and the administration of AGPs such as chlortetracycline and sulfamethazine (Aureo S-700 G) is the standard. The impacts of AGPs on the intestinal microbiota of beef cattle are currently uncertain; it is documented that AGPs administered to beef cattle pass through the rumen and enter the intestine. To ascertain the impacts of Aureo S-700 G on the small and large intestinal microbiota of beef cattle (mucosa-associated and within digesta), terminal restriction fragment length polymorphism (T-RFLP) analysis and quantitative PCR (qPCR) for total bacteria were applied. Beef cattle were maintained in an experimental feedlot (five replicate pens per treatment), and AGP treatment cattle were administered Aureo S-700 G in feed, whereas control cattle were administered no antimicrobials. As the intestinal microbiota of beef cattle has not been extensively examined, clone library analysis was applied to ascertain the primary bacterial constituents of the intestinal microbiota.

**Results:**

Comparative T-RFLP and qPCR analysis (n = 122 samples) revealed that bacterial community fingerprints and bacterial load within digesta differed from those associated with mucosa. However, the administration of Aureo S-700 G did not affect bacterial community fingerprints or bacterial load within the small and large intestine relative to control cattle. Analysis of >1500 near full length 16S rDNA clones revealed considerably greater bacterial diversity in the large relative to the small intestine of beef cattle. Mucosa-associated bacterial communities in the jejunum were dominated by Proteobacteria, and differed conspicuously from those in the ileum and large intestine. Although the ileum contained bacterial clones that were common to the jejunum as well as the cecum, Firmicutes clones associated with mucosa dominated in the ileum, cecum, and descending colon. In the descending colon, clone library analysis did not reveal a difference in the richness or diversity of bacterial communities within digesta relative to those associated with mucosa. However, T-RFLP analysis indicated a significant difference in T-RF relative abundance (i.e. difference in relative taxon abundance) between mucosa-associated and digesta communities attributed in part to the differential abundance of *Bacteriodes*, *Alistipes*, *Oscillibacter*, and unclassified Clostridiales.

**Conclusions:**

These data demonstrate that there was no significant difference in the composition of the predominant intestinal bacteria constituents within animals administered Aureo S-700 G and those not administered AGPs after a 28 day withdrawal period.

## Background

Antimicrobial growth promoters (AGPs) are commonly administered in non-therapeutic concentrations in/on the livestock feed to increase animal weight gain per unit of feed consumed. Concerns over the development of antimicrobial resistance (AMR) and the potential transmission of resistant zoonotic pathogens to humans have become prominent societal issues, and have resulted in a ban on AGP use within the European Union (EU) [1,2]. The AGP ban within the EU has concurrently increased the use of therapeutically administered antimicrobials [1] and the cost of animal production [3]. A recent guidance document issued by the United States Food and Drug Administration recommended restrictions that will limit the use of AGPs [4]. Thus, it is anticipated that an AGP ban will progressively be imposed in North America.

The impending loss of AGPs in North America has precipitated a renewed interest in identifying efficacious alternatives to AGPs. However, a current lack of knowledge on the mechanisms by which AGPs function has hampered the development of alternatives. The literature on the mode of action of AGPs is scarce; however, the most widely accepted hypothesis is that AGPs modulate the intestinal microbiota [5,6]. The ‘microbiota modulation’ hypothesis suggests that AGPs reduce microbial competition for nutrients, decrease production of growth depressing metabolites by intestinal microorganisms, suppress opportunistic pathogens, and result in a thinner intestinal wall, which increases nutrient assimilation [5]. The consistency of growth promotion effects imparted by AGPs on various animal species possessing highly dissimilar intestinal microbiota, coupled with the low concentrations at which AGPs are administered (i.e. at doses less than the minimum inhibitory concentration for most pathogens) questions the validity of the microbiota modulation hypothesis of AGP action [7]. Furthermore, the high prevalence of carriage of antimicrobial resistance determinants by livestock in the absence of selection pressure [8] is not consistent with the microbiota modulation hypothesis. Many antimicrobials have anti-inflammatory and immunomodulatory properties [9], and we have recently shown that the administration of chlortetracycline administered at non-therapeutic concentrations modulated the enteric immune response, suggesting that AGPs may function by suppressing inflammatory responses within the intestine thereby providing a catabolic advantage to the host (i.e. the ‘immunodulation’ hypothesis for AGP action) [10].

Beef cattle are a significant livestock species produced in North America, and the administration of AGPs to beef cattle in confined feeding operations (i.e. feedlots) is the industry standard [11]. AGPs administered to cattle pass through the rumen into the intestine, and are excreted in feces [12,13]. Yet characterization of the intestinal microbiota of cattle, including the impacts of AGPs on the microbiota has received very limited attention. Consistent with the immunomodulation hypothesis of AGP action [10], we erected the hypothesis that AGPs administered to beef cattle will not affect the intestinal microbiota. Using animals maintained in an experimental feedlot, the study objectives were to characterize the intestinal microbiota of cattle, and statistically contrast bacterial community fingerprints and loads in the small and large intestine of cattle administered Aureo S-700 G relative to animals not administered an AGP.

## Results

### T-RFLP community analysis

**Figure 1:**
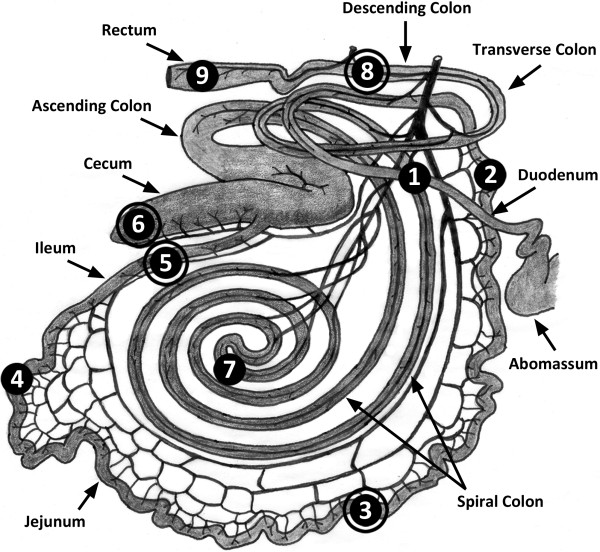
**Bovine intestine recreated from Nickel *et al*. **[14]**.** Black circles indicate mucosal locations sampled and processed, and outlined circles indicate locations where digesta was sampled and processed in addition to mucosa. Sample locations were: (**1**) descending portion of the duodenum; (**2**) proximal jejunum; (**3**) central jejunum; (**4**) distal jejunum; (**5**) ileum; (**6**) free end of the cecum; (**7**) central flexure of the ascending colon; (**8**) descending colon; and (**9**) rectum.

Mucosal samples from nine intestinal locations of ten steers (i.e. 90 samples), and digesta samples from the central jejunum, ileum, cecum, and descending colon were processed (Figure 1). It was not possible to obtain adequate digesta from all animals at all intestinal locations; however, 32 digesta samples (out of a possible 40) were processed. Digesta samples were obtained from the central jejunum and ileum of three control treatment cattle, from the ileum of three AS700 treatment cattle, and from the cecum and descending colon of four AS700 treatment cattle.

**Figure 2:**
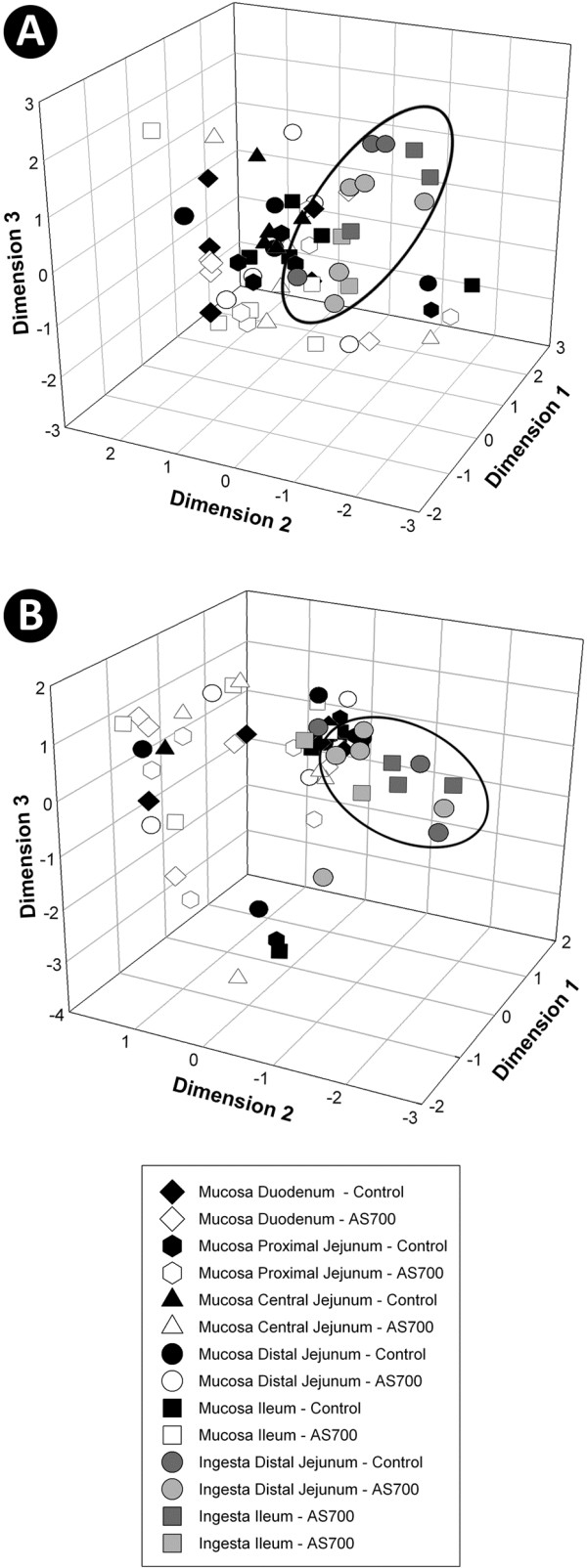
**Non-metric multi-dimensional scaling plots.** Plots depict community terminal restriction fragment (T-RF) presence/absence (**A**) and T-RF relative abundance (**B**) within the small intestine of control and AS700 treatment cattle. All locations consist of five replicate animals, with the exception of digesta from the distal jejunum for control treatment cattle (n = 3), and from the ileum of control and AS700 treatment cattle (n = 3). Each marker represents a bacterial community for one sample. Ellipsoids represent predominant clustering of digesta relative to mucosa-associated communities.

**Figure 3:**
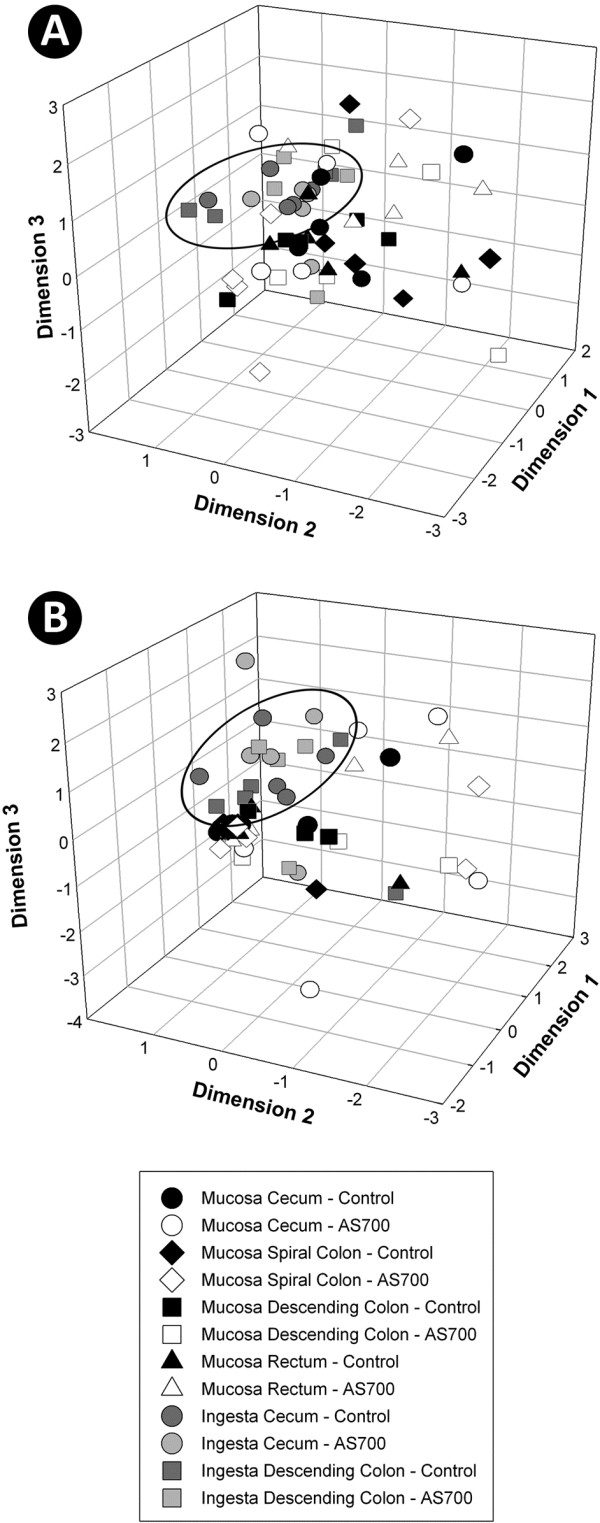
**Non-metric multi-dimensional scaling plots.** Plots depict community terminal restriction fragment (T-RF) presence/absence (**A**) and T-RF relative abundance (**B**) within the large intestine of control and AS700 treatment cattle. All sections consist of five replicate animals, with the exception of digesta from the cecum and descending colon for cattle administered AS700 (n = 4). Each marker represents a bacterial community for one sample. Ellipsoids represent predominant clustering of digesta relative to mucosa-associated communities.

Diverse bacterial communities were observed in association with the intestinal mucosa and digesta at all nine intestinal locations. Total numbers of T-RFs by sample location ranged from 96 to 303 (Table 1). For both digesta and mucosa samples, intestinal location affected (P ≤ 0.025) the number of T-RFs; in general, fewer T-RFs (P ≤ 0.050) were observed in the small intestine relative to the large intestine. Based on presence/absence of T-RFs, bacterial community fingerprints associated with mucosa differed (P ≤ 0.001) from those within digesta in the small and large intestine (Table 2). Consistent with the group significance test results, non-metric multidimensional scaling (NMS) plots showed that bacterial communities within digesta clustered separately from communities associated with mucosa in the small (Figure 2A) and large (Figure 3A) intestines. Differences in community fingerprints associated with mucosa relative to digesta based on T-RF relative abundance were also observed in the small and large intestine (P ≤ 0.001), as well as for all individual locations (P ≤ 0.036) (Table 2). Similarly, NMS plots showed that bacterial communities within digesta clustered separately from communities associated with mucosa (Figure 2B, 3B). It is important to emphasize that different Qiagen kits were used to extract genomic DNA from digesta versus mucosa. Although these kits are both commonly used to extract bacterial genomic DNA for community analyses, they have never been comparatively evaluated to our knowledge. Thus, it is possible that the conspicuous differences observed in community composition between the two substrates were influenced by the extraction method.

**Table 1:** **Mean terminal restriction fragment (T-RF) number for digesta and mucosal samples obtained from beef cattle administered (AS700) or not administered antimicrobial growth promoters (Control)**^**a**^

**Sample/location**	**AS700**	**Control**	**LSD**^**b**^	**Total**^**c**^
Digesta				
Central jejunum	57.2 ± 2.4^d^	45.3 ± 8.6	a	153
Ileum	78.5 ± 10.5	52.3 ± 8.5	ab	254
Cecum	89.0 ± 7.7	80.2 ± 8.1	d	303
Descending colon	67.3 ± 3.9	68.4 ± 5.2	bc	247
Duodenum	51.8 ± 6.4	53.2 ± 11.2	ab	96
Proximal jejunum	55.6 ± 7.0	58.0 ± 5.2	abc	166
Central jejunum	57.4 ± 8.4	46.2 ± 8.2	a	156
Distal jejunum	46.6 ± 10.7	52.6 ± 6.3	a	181
Ileum	60.6 ± 13.6	54.8 ± 8.5	abc	142
Cecum	79.6 ± 10.7	79.4 ± 12.1	d	216
Spiral colon	57.6 ± 7.3	67.4 ± 17.9	abcd	195
Descending colon	60.6 ± 13.2	87.4 ± 6.9	cd	267
Rectum	62.8 ± 7.9	80.8 ± 10.4	bcd	236

^a^ All samples consisted of five replicate cattle per treatment, with the exception of digesta from the central jejunum of treatment cattle (n = 3), from the ileum of control and AS700 treatment cattle (n = 3), and from the cecum and descending colon of AS700 treated cattle (n = 4).

^b^ Within each sample type (i.e. digesta or mucosa), locations (averaged over treatment) not followed by the same letter differ (P ≤ 0.05) as determined by Fisher's least significant difference (LSD).

^c^ Total unique T-RFs (all animals combined by location and sample type).

^d^ Values represent the mean ± the standard error of the mean.

**Table 2:** **Group significance pairwise comparisons of T-RFLP bacterial community fingerprints associated with mucosa or within digesta between beef cattle administered (AS700) or not administered antimicrobial growth promoters (Control)**^**a**^

**Treatment/location**	**T-RF presence/absence**		**T-RF relative abundance**	
	**Mucosa**	**Digesta**	**Mucosa**	**Digesta**
Treatments combined				
Small intestine	0.001**	0.001**	0.001**	0.001**
Large intestine	0.001**	0.001**	0.001**	0.001**
All samples	0.001**	0.001**	0.001**	0.001**
AS700				
Central jejunum	0.504	0.002**	0.001**	0.001**
Ileum	0.484	0.002**	0.028*	0.036*
Cecum	0.707	0.006**	0.571	0.007**
Descending colon	0.780	0.013*	0.002**	0.001**
Control				
Central jejunum	0.363	0.144	0.001**	0.001**
Ileum	0.100	0.087	0.001**	0.001**
Cecum	0.433	0.040*	0.001**	0.001**
Descending colon	0.225	0.064	0.001**	0.001**

^a^ All samples consisted of five replicate animals per treatment, with the exception of digesta from the central jejunum of treatment cattle (n = 3), from the ileum of control and AS700 treatment cattle (n = 3), and from the cecum and descending colon of AS700 treated cattle (n = 4). The two p-values presented per comparison reflect inter-group variability.

*Significant difference (P ≤ 0.05 and P > 0.01) between mucosa and digesta by treatment/location for each of T-RF presence and T-RF relative abundance (i.e. mucosa compared to digesta and visa-versa).

**Highly significant difference (P ≤ 0.01) between mucosa and digesta.

**Figure 4:**
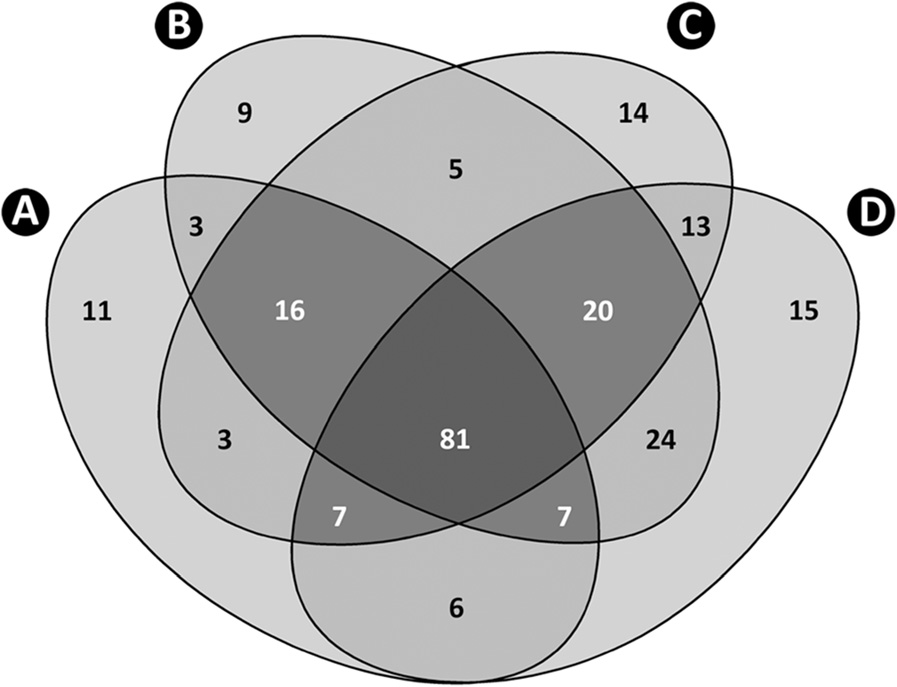
**Four-way Venn diagram.** Unique and common T-RFs among the small and large intestines of beef cattle not administered (control treatment) and administered antimicrobial growth promoters (AS700 treatment) are shown where: (**A**) small intestine of control treatment cattle; (**B**) large intestine of control treatment cattle; (**C**) small intestine of AS700 treatment cattle; and (**D**) large intestine of AS700 treatment cattle.

Administration of AS700 did not affect (P = 0.346) numbers of T-RF associated with mucosa by sample location. However, more (P = 0.034) T-RFs were observed in digesta from cattle that ingested AS700 relative to control treatment cattle (i.e. averaged across location) (Table 1). With the exception of mucosa-associated communities in the spiral colon (P = 0.047), AGP administration did not affect (P > 0.050) the community fingerprints (based on T-RF presence/absence) associated with mucosa or within digesta of the small and large intestine (Table 3). Similarly, minimal differences between the control and AS700 treatment were observed based on T-RF relative abundance; however, mucosa-associated communities in the proximal and central jejunum, and cecum differed slightly (P < 0.03) between the two treatments. Consistent with pairwise results, AS700 treatment cattle community fingerprints did not cluster separately from control treatment cattle in NMS plots for both mucosa-associated (Figure 2A, 3A) and digesta (Figure 2B, 3B) communities. Examination of T-RFs showed that the majority were common to control and AS700 treatment cattle within the small (56.6%, n = 107) and large (64.1%, n = 132) intestine (Figure 4).

**Table 3:** **Group significance pairwise comparisons of T-RFLP bacterial community fingerprints associated with mucosa or within digesta between beef cattle administered (AS700) or not administered antimicrobial growth promoters (Control)**^**a**^

**Sample/location**	**T-RF presence/absence**		**T-RF relative abundance**	
	**AS700**	**Control**	**AS700**	**Control**
Digesta				
Central jejunum	0.237	0.540	0.571	0.150
Ileum	0.718	0.140	0.588	0.169
Cecum	0.502	0.627	0.541	0.158
Descending colon	0.419	0.602	0.578	0.127
Mucosa				
Duodenum	0.667	0.745	0.544	0.166
Proximal jejunum	0.852	0.269	0.335	0.030*
Central jejunum	0.872	0.062	0.919	0.009**
Distal jejunum	0.922	0.742	0.738	0.879
Ileum	0.324	0.409	0.321	0.066
Cecum	0.881	0.281	0.918	0.001**
Spiral colon	0.047*	0.462	0.468	0.062
Descending colon	0.959	0.167	0.954	0.350
Rectum	0.810	0.115	0.742	0.347

^a^ All samples consisted of five replicate animals per treatment, with the exception of digesta from the central jejunum of treatment cattle (n = 3), from the ileum of control and AS700 treatment cattle (n = 3), and from the cecum and descending colon of AS700 treated cattle (n = 4). The two p-values presented per comparison reflect inter-group variability.

*Significant difference (P ≤ 0.05 and P > 0.01) between the AS700 and control treatments by sample type/location for each of T-RF presence/absence and T-RF relative abundance (i.e. AS700 compared to control and visa-versa).

**Highly significant difference (P ≤ 0.01) between the AS700 and control treatments by sample type / location.

### Quantitative PCR

**Figure 5:**
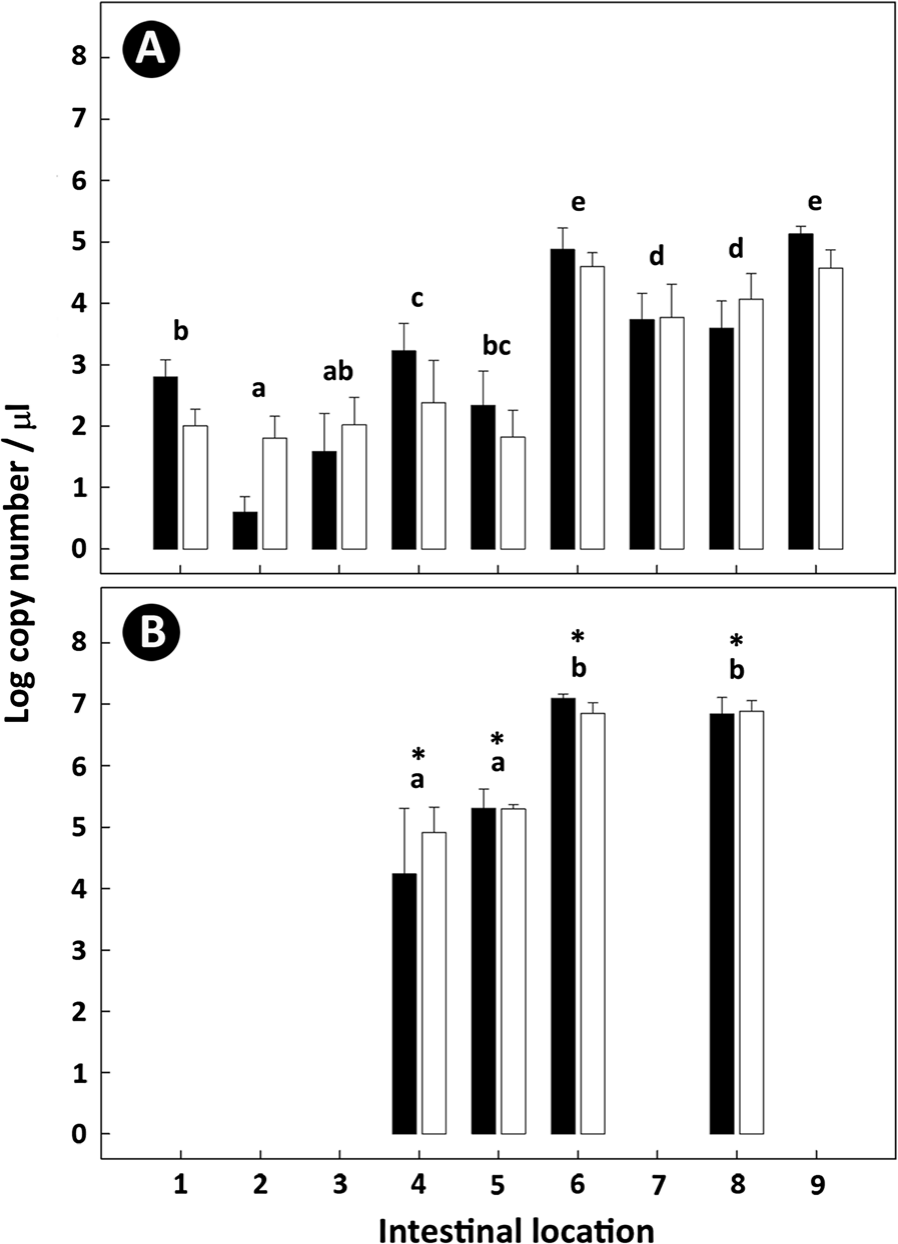
**Relative bacterial densities throughout the intestine of beef cattle.** Bacterial densities associated with mucosa (**A**) or within digesta were measured in the small and large intestine of beef cattle (log_10_ copy number μl^-1^ of template 16S rDNA). Treatments are cattle administered AS700 (black histograms) and cattle not administered antimicrobials (white histograms). Intestinal locations are: (1) duodenum; (2) proximal jejunum; (3) central jejunum; (4) distal jejunum; (5) ileum; (6) cecum; (7) spiral colon; (8) descending colon; and (9) rectum. Vertical lines associated with histograms indicate the standard error of the mean. Within **A** and **B**, histogram bars at each location not followed by the same letter differ (P < 0.05). Bars indicated by an asterisk differ (P < 0.001) between digesta and mucosa-associated at corresponding locations.

Densities of total bacteria associated with mucosa or within digesta did not differ between the AS700 and control treatments (P ≤ 0.66) (Figure 5A). However, differences in densities of mucosa-associated bacteria were observed in the different regions of the intestine. Less (P ≤ 0.026) bacteria were present in the small relative to the large intestine. Within the small intestine, higher densities (P ≤ 0.042) of bacteria occurred in the duodenum than in the proximal small intestine. The highest densities (P ≤ 0.035) of mucosa-associated bacteria occurred in the cecum and rectum. Bacterial densities in digesta were 1.7 to 3.0 orders of magnitude larger (P < 0.001) than the corresponding mucosal samples (Figure 5A-B). Bacterial numbers in digesta within the small intestine were smaller (P < 0.001) than in the large intestine (Figure 5B). There was no difference in bacterial densities within digesta in the cecum or descending colon.

### Clone library community analysis

**Figure 6:**
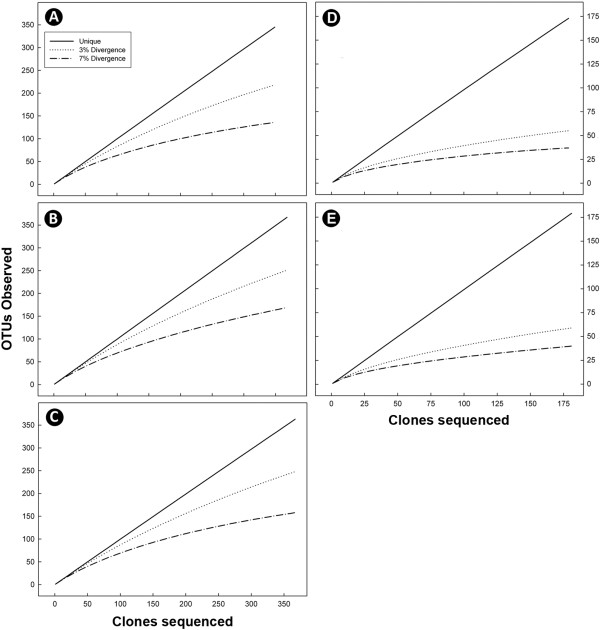
**Rarefaction curves.** Rarefaction analysis was performed on: (**A**) mucosa-associated communities in the cecum (n = 349); (**B**) mucosa-associated communities in the descending colon (n = 368); (**C**) communities within digesta in the descending colon (n = 367); (**D**) mucosa-associated communities in the central jejunum (n=179); and (**E**) mucosa-associated communities in the ileum (n = 181).

**Figure 7:**
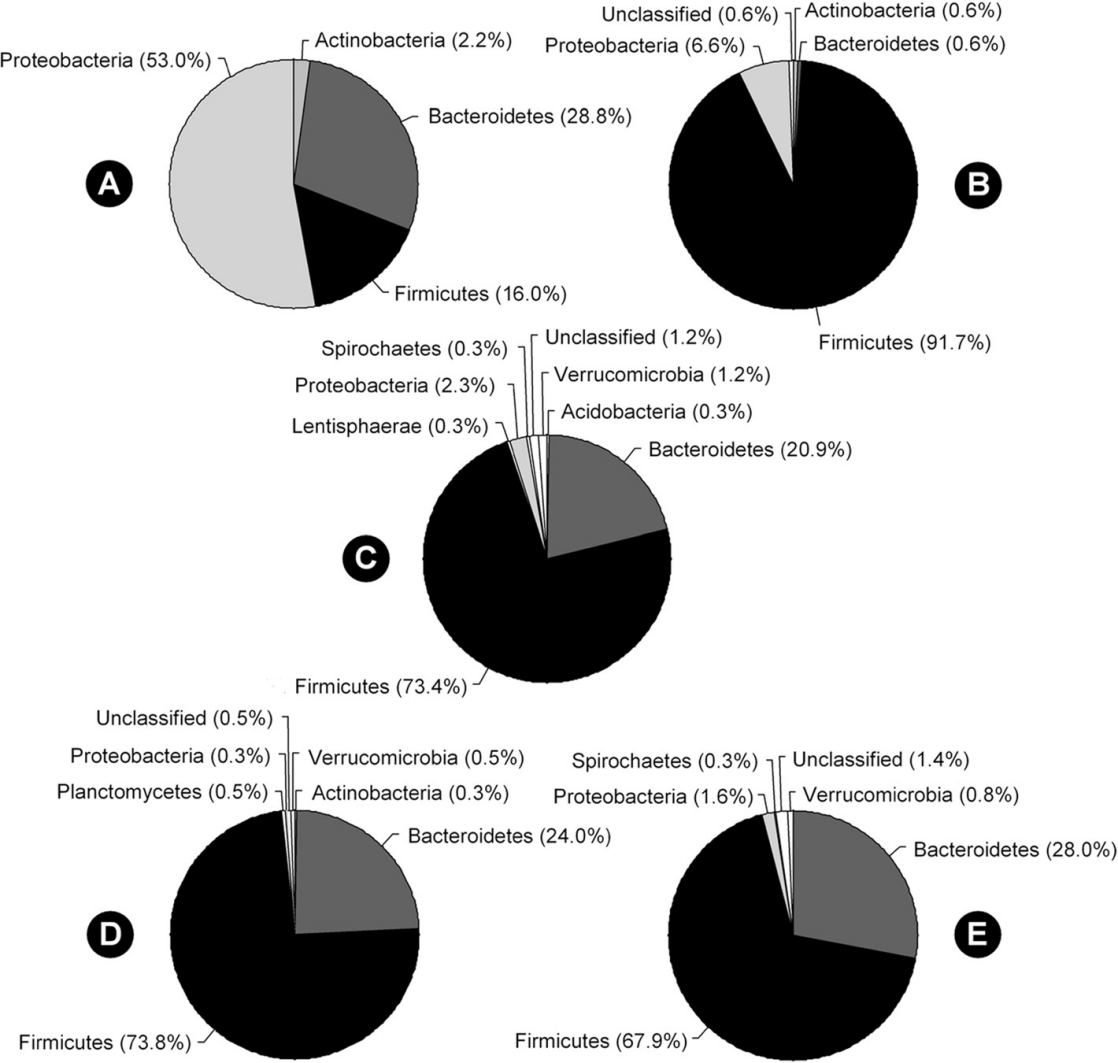
**Phylum level classification.** Classifications were determined using RDP classifier (16S rRNA gene sequences) for: (**A**) mucosa-associated communities in the central jejunum (n = 179 total sequences); (**B**) mucosa-associated communities in the ileum (n = 181); (**C**) mucosa-associated communities in the cecum (n = 349); (**D**) mucosa-associated communities in the descending colon (n = 368); (**E**) communities within digesta in the descending colon (n = 367).

**Figure 8:**
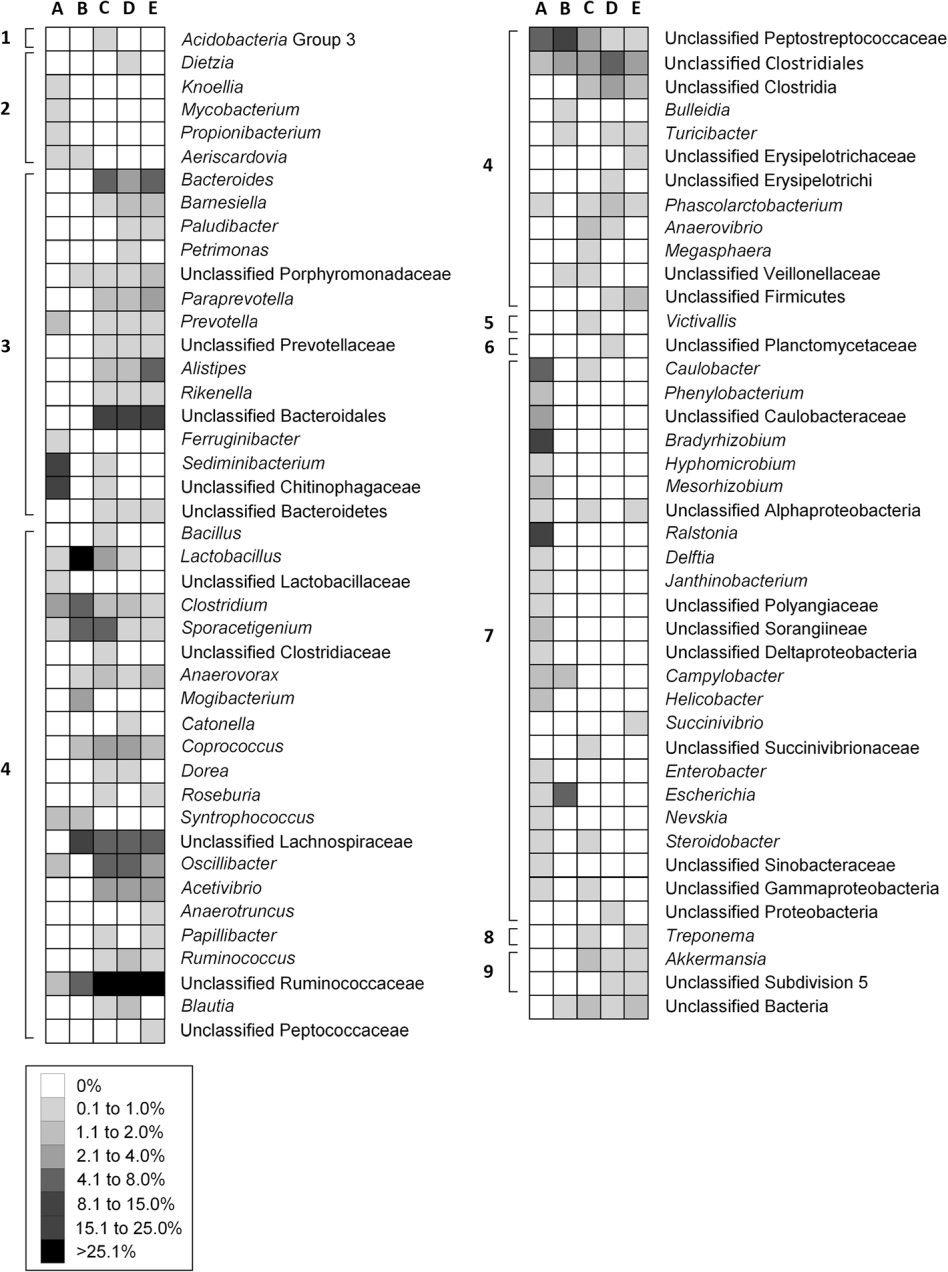
**Non-linear heat map of 16S rRNA gene clone frequencies.** Frequencies are presented for: (**A**) mucosa-associated communities in the central jejunum; (**B**) mucosa-associated communities in the ileum; (**C**) mucosa-associated communities in the cecum; (**D**) mucosa-associated communities in the descending colon; and (**E**) communities within digesta in the descending colon. Clones were assigned to the following phyla: (1) Acidobacteria; (2) Actinobacteria; (3) Bacteroidetes; (4) Firmicutes; (5) Lentisphaerae; (6) Planctomycetes; (7) Proteobacteria; (8) Spirochaetes; and (9) Verrucomicrobia.

Considerable bacterial diversity was associated with the mucosa of the small and large intestine, as well as within digesta of the descending colon of beef cattle (Table 4). However, greater community richness and diversity were observed in the large intestine relative to the small intestine. Twice as many clones from the large intestine were sequenced in comparison to the small intestine, yet the restricted sampling within the small intestine better reflected the diversity of operational taxonomic units (OTUs) associated with mucosa (Figure 6). The composition of the bacterial community associated with mucosa of central jejunum differed from that of the ileum, cecum and descending colon (Figure 7); communities within the central jejunum contained more Proteobacteria clones, specifically *Ralstonia*, *Bradyrhizobium*, and *Caulobacter*, while the ileum, cecum and descending colon were dominated by Firmicutes clones (Figure 8; Additional file 1: Figure S1, Additional file 2: Figure S2, Additional file 3: Figure S3, Additional file 4: Figure S4). *Lactobacillus*, and unclassified Lachnospiraceae, Ruminococcaceae and Peptostreptococccus clones were the most prevalent Firmicutes in the ileum, while unclassified Lachnospiraceae and Ruminococcaceae clones were the most prevalent Firmicutes in the large intestine. Bacteria within the phylum Bacteriodetes were less common in association with the mucosa in the ileum (0.6%) relative to the large intestine (>20.9%). Numbers of predicted OTUs associated with mucosa were 55, 59, 218, and 251 in the central jejunum, ileum, cecum and descending colon, respectively, and 248 from digesta within the colon (Table 4). In comparison, total T-RFs observed in the corresponding samples numbered 46.2 ± 8.2 (156 total), 54.8 ± 8.5 (142 total), 79.4 ± 12.1 (216 total), 87.4 ± 6.9 (267 total), and 68.4 ± 5.2 (247 total) T-RFs, respectively (Table 1). Richness, diversity, and community composition of bacteria associated with mucosa relative to within digesta did not differ within the descending colon (Table 4; Figure 8; Additional file 4: Figure S4, Additional file 5: Figure S5). This is consistent with the observation of no difference in T-RF presence/absence (Table 2; Figure 2–3).

**Table 4:** Operational taxonomic units (OTUs) observed and community richness and diversity indices (97% DNA sequence similarity)

	**Community richness**			**Community diversity**		
**Sample**	**OTUs**	**Chao**	**ACE**	**Shannon’s**	**Non-parametric Shannon’s**	**Simpson’s**
Central jejunum ­ mucosa [179]^a^	55	95	145	3.41	3.70	0.0455
		(71–155)^b^	(108–207)^b^	(3.25-3.58)^b^		(0.0349-0.0560)^b^
Ileum ­ mucosa [181]	59	122	240	3.30	3.65	0.0766
		(86-211)	(184-320)	(3.10-3.51)		(0.0494-0.104)
Cecum ­ mucosa [349]	218	430	533	5.17	5.80	0.00492
		(355-548)	(418-713)	(5.07-5.27)		(0.00335-0.00650)
Descending colon ­ mucosa [368]	251	663	651	5.38	6.09	0.00287
		(520–883)	(521–842)	(5.29-5.46)		(0.00211-0.00364)
Descending colon ­ digesta [367]	248	607	644	5.36	6.06	0.00305
		(483–795)	(517–833)	(5.27-5.44)		(0.00223-0.00388)

^a^Values in square brackets indicate number of clones.

^b^Values in parentheses indicate 95% confidence intervals.

## Discussion

Although considerable effort has been expended characterizing microbial communities in the rumen of cattle, very limited research has focused on characterizing the microbiota within the bovine intestine. In the current study, we used T-RFLP in conjunction with qPCR and clone library analyses to examine bacterial communities associated with mucosa and within digesta throughout the intestinal tract of beef cattle not previously exposed to antimicrobials at any point in their lives. We observed that community fingerprints differed along the intestinal tract. In particular, greater T-RF diversity was observed in the large intestine relative to small intestine. In monogastric animals, both the microbial load and species diversity significantly increase in the ileum and throughout the colon [15].

Mucus secreted from goblet cells forms two distinct layers in the gastrointestinal (GI)-tract [16]. These layers vary in thickness throughout the intestine, and evidence indicates that bacteria readily colonize the loosely adherent mucus layer but not the adherent mucus layer [17]. The bacteria that colonize this mucus layer are likely important in maintaining host health [18,19]. We observed that community fingerprints associated with mucosa differed from digesta in adjacent locations for all intestinal locations sampled. This observation is consistent with a recent finding in bovine calves using denaturing gradient gel electrophoresis [20]. Almost all characterizations of the intestinal microbiota of cattle utilizing molecular methods to date have focused on the examination of fecal matter [21-23]. However, the fecal microbiota is not necessarily representative of the intestinal microbiota [24]. Fecal sampling is also limiting in that it does not allow an examination of localized communities within the intestine (i.e. autochthonous bacteria). This is further complicated by the release of autochthonous bacteria from proximal regions of the GI-tract, such as the rumen, that survive transit.

To characterize the composition of the intestinal microbiota of beef cattle, we completed traditional clone analysis (near complete 16S rRNA gene sequence) of composite samples obtained from the central jejunum, ileum, cecum and descending colon; community fingerprints of the samples were determined not to differ significantly by T-RFLP analysis prior to pooling. Proteobacteria clones were conspicuously more abundant (53% of clones sequenced) in the jejunum compared to the ileum (6.6%), cecum (2.3%), and descending colon (0.3%). Proteobacteria are abundant in the rumen [25], but to our knowledge the genera that we detected are not recognized as abundant rumen constituents. While the ileum contained bacterial clones that were common to the jejunum as well as the cecum, Firmicutes clones associated with mucosa dominated in both the ileum (91.7%) and cecum (73.4%). *Lactobacillus* and *Streptococcus* have been readily isolated throughout the bovine intestinal tract [26,27]. Although we did not detect any *Streptococcus* clones, we did detect *Lactobacillus* clones at all sites with a particular predominance in mucosa-associated samples within the ileum. The cecum of monogastric animals is dominated by obligate anaerobes, and it is currently unclear whether the ileal microbiota represents a unique community or these bacteria emanate from the cecum via leakage of cecal bacteria through the ileocecal valve [15]. Although we did not detect either *Bacteroides* or *Prevotella* clones in the ileum as compared to the cecum, our data tended to support a cecal origin given that common taxa and taxonomic groups were observed in the ileum and the cecum, yet the richness and diversity of bacteria communities in the ileum were much lower. Although bacteria in the ileum likely originate from the cecum, their association with with mucosa suggests that they are autochthonous within the ileum. Despite its prominence, the nutritional importance of the cattle cecum and its possible role as a reservoir of enteric bacteria has not been fully determined.

An antimicrobial agent is a growth promoter when administered at non-therapeutic concentrations in/on the feed of food animals to promote growth and enhance feed efficiency. Limited research has investigated the impacts of these non-therapeutic concentrations of antimicrobials on the enteric microbiota. Of note, chlortetracycline and sulfamethazine administered at non-therapeutic concentrations pass through the rumen of cattle into the intestine, and are subsequently excreted in feces [12,13]. Furthermore, a significant amount of the chlortetracycline (administered as Aureo AS700 G) is excreted in beef cattle feces in its non-isomerized form [13], which is microbiologically active [28]. Selection for enteric pathogens and pathogen surrogates resistant to antimicrobials following administration of AGPs has promulgated the belief that AGPs induce growth promotion in livestock by their direct effects on the intestinal microbiota (i.e. the microbiota modulation hypothesis) [5,29]. To contrast intestinal bacterial communities in cattle administered an AGP relative to control treatment animals, we used T-RFLP analysis. Data showed that the oral administration of chlortetracycline and sulfamethazine at non-therapeutic levels to cattle for 193 days did not appreciably affect the intestinal microbiota associated with mucosa or within digesta in either the small or large intestine relative to cattle not administered antimicrobials. T-RFLP is a medium- to high-throughput comparative community fingerprinting method and is recognized to provide both high resolution and reproducible results [30] that are comparable to pyrosequence-based analysis of communities [31]. The T-RFLP method has been criticized by some as it does not provide the depth of coverage of pyrosequencing. However, the biological-relevance of rare occurring OTU is uncertain, and T-RFLP analysis showed no significant impact of AS700 on the predominant constituents of the intestinal microbiota of beef cattle. Furthermore, the utilization of T-RFLP analysis allowed us to process a large number of samples (n = 122) without the high cost of next-generation sequencing. This is particularly important in light of recent evidence indicating that analysis of more samples at the expense of sequence coverage is recommended [32], and that a reliance on pseudo-replicates greatly increases the chances of committing a type I statistical error [33].

Our results are in agreement with the conclusions of Kalmokoff *et al*. [8] and McGarvey *et al*. [23], but contradict those of Rettedal *et al*. [34], Looft *et al*. [35], and Kim *et al*. [36]. Kalmokoff *et al*. [8], McGarvey *et al*. [23], and Rettedal *et al*. [34] utilized clone library analysis, whereas Looft *et al*. [35] and Kim *et al*. [36] used pyrosequence-based analysis of bacterial communtities. Kalmokoff *et al*. [8] showed that continuous administration of non-therapeutic erythromycin to pigs did not affect fecal community structure, and McGarvey *et al*. [23] observed that the administration of the ionophore, monensin, did not alter the fecal microbiota in cattle. In contrast, Kim *et al*. [36] concluded that tylosin phosphate administered to pigs altered the composition of the bacterial community in feces. Of note, pyrosequencing was used in this study, but considerable inter-animal variability by farm and sample date was observed, thereby limiting the author’s ability to definitively conclude that tylosin contributed to growth promotion via modulation of the intestinal community. Utilizing pyrosequence-based analysis, Looft *et al*. [35] also concluded that chlortetracycline, sulfamethazine, and penicillin administered to piglets at non-therapeutic concentrations for only 14 days altered the microbiota, namely by increasing the prevalence of Proteobacteria. However, they administered antimicrobials for a very short duration, a small number of animals were examined once, and analyses were restricted to feces. Furthermore, Proteobacteria are not abundant constituents of the colonic microbiota. Based on a critical review of the AGP literature, Niewold [7] concluded that cumulative evidence does not support the commonly held belief that AGPs primarily function by modulating the intestinal microbiota. It is recognized that many antimicrobials administered at therapeutic concentrations are capable of altering immune responses and bacterial communities within the intestine [9,37]. However, Costa *et al*. [10] recently showed that chlortetracycline administered to mice at non-therapeutic concentrations modulated enteric inflammation in the absence of conspicuous effects on the microbiota. It is likely that the mode of action of AGPs is complex and involves an interactive effect on the host and the intestinal microbiota.

Similarly to other species, a high degree of inter-animal variability occurs in beef cattle [22]. Our experimental design allowed us to treat animals identically with the exception that AGP treatment cattle were administered AS700 daily for 193 days (maximum exposure model), whereas control treatment cattle were not administered an AGP. A strength of our study is that experimental units (i.e. individual animals) represented true replicates thereby allowing us to obtain a measure of variability independent from treatment effects. In this regard, treatment animals (100 beef cattle) were placed in 10 separate pens, and one animal was arbitrarily selected per pen and evaluated. Furthermore, cattle were maintained in an experimental feedlot for ca. 8 months to replicate an actual production scenario. However, the administration of AS700 ended 28 days before animals were euthanized and samples were obtained (i.e. to meet mandatory withdrawal requirements for AGPs). Thus, we are unable to definitely ascertain whether the microbiota reverted to baseline in the absence of selection pressure during the AGP withdrawal period. This possibility appears unlikely given that differential carriage of tetracycline and sulfamethazine resistance determinants occurred in the fecal bacterial community of AS700 versus control treatment cattle, and carriage rates did not change after antimicrobial administration halted (data not presented). None-the-less, the impact of AGP withdrawal on intestinal community structure and carriage of AGP resistant determinants warrants investigation in subsequent research.

## Materials and methods

### Animals

Beef cattle were housed in an experimental feedlot located at the Lethbridge Research Centre. Animals originated from a common location and did not receive any antimicrobials before the initiation of the experiment. Animals were arbitrarily assigned to one of two treatments: (i) no antimicrobials (control treatment) or (ii) 350 mg head^-1^ day^-1^ chlortetracycline and 350 mg head^-1^ day^-1^ sulfamethazine (Aureo S 700 G; Alpharma Inc., NJ) [AS700 treatment]. Aureo S 700 G is a commonly used AGP for beef production in North America [38], and was fed at a non-therapeutic concentration as recommended by the manufacturer. Each treatment was replicated five times, and the treatment groups were arranged in a complete randomized design; each replicate consisted of a separate pen containing ten steers. Water troughs were shared between adjacent pens, but arranged in a manner so that only cattle that received AS700 could drink from the same water trough.

Before commencement of the study, the experiment was approved by the Lethbridge Research Centre Animal Care Committee, and all cattle involved in this study were cared for according to the guidelines set out by the Canadian Council on Animal Care [39]. Steers entering the feedlot November 29, 2004 were fed a forage based diet consisting of 70% barley silage, 25% barley grain, and 5% (dry matter basis) supplement with vitamins and minerals for the first 84 days (i.e. ‘backgrounding’ period). Cattle were subsequently transitioned from the predominately forage based diet to a predominantly grain based diet over a 21 day period, and then maintained on the grain based diet (85% barley, 10% barley silage, 5% supplement) for an additional 126 days (i.e. ‘finishing’ period); this feeding regimen is typical for the Canadian feedlot industry. Cattle were fed once daily in a manner that ensured that all feed that was allocated to each pen was consumed. AS700 was first introduced into the diets 5 days after the cattle arrived at the feedlot, and it was included in the diet for 193 days thereafter; AS700 was removed from the diet 28 days prior to slaughter to meet the requisite withdrawal period. To avoid cross contamination, antimicrobials were mixed with 5 kg of a supplement containing minerals and vitamins, and the mixture was manually spread over the surface of feed in each of the appropriate pens; all cattle in the pen were capable of feeding at the feed trough at the same time. Supplement that did not contain any antimicrobials was spread on the feed of cattle assigned to the control treatment.

### Sample collection

Fecal samples were obtained from cattle upon arrival at the feedlot and at intervals thereafter (total of nine sample times over a 226-day period). To obtain samples, individual cattle were placed in constraint device, and fecal samples were extracted per rectum. Care was taken to ensure that animals did not cross contaminate each other across treatments. Fecal samples were immediately placed on ice, and within 1 h of collection samples were transported to the laboratory and stored at −20°C until processed.

For mucosa and digesta samples, one animal per replicate from each of the control and AS700 treatment was randomly selected for sampling in the abattoir (n = 5 cattle per treatment); the abattoir used was a provincially inspected medium capacity plant. Cattle were transported to the abattoir on the evening prior to euthanization. Control treatment cattle were transported on July 17, 2005 and slaughtered the next day, whereas AS700 cattle were transported July 18, 2005 and also slaughtered the next day. Following transport of the control treatment cattle, the stock trailer was thoroughly cleaned using a pressure washer. At the abattoir, cattle were maintained on a barley silage diet, and were euthanized humanely according to the Canadian Council on Animal Care [39]. The intestinal tract of individual cattle was removed ≈ 10 min after euthanization, and placed on a clean sheet of plastic on a cool cement floor within the abattoir. Nine intestinal sections (≈20-cm long) were obtained from each animal at the following locations: descending portion of the duodenum (i.e. following the cranial flexure); proximal jejunum; central jejunum; distal jejunum; ileum (≈10 cm before the ileal-cecal junction); free end of the cecum; spiral colon (i.e. central flexure of the ascending colon); descending colon (≈20 cm before the sigmoid colon); and rectum. Before excision of the intestinal sections, bilateral ligatures were applied adjacent to the excision site to minimize external contamination of the tissues with digesta. Tissue samples were then placed in individual bags on ice and transported to the laboratory for processing.

Mucosa and digesta within the intestinal lumen were sampled. Intestinal sections were aseptically excised longitudinally and digesta removed aseptically. Digesta from each sample was weighed (200 mg ± 20 mg) and placed in DNA free tubes. Following removal of the majority of digesta, the mucosal surface within each intestinal section was gently washed with chilled sterile phosphate buffer with saline (PBS; 10 mM sodium phosphate buffer with 130 mM sodium chloride; pH 7.2) taking care to remove residual digesta while minimizing disruption of mucus on the mucosal surface. Mucosal sections were removed with a sterile 4-mm diameter Biopsy Acu Punch (CDMV, St. Hyacinthe, QC) and individual plugs were placed in DNA free tubes. Digesta and mucosal plugs were stored at −20°C until processed.

### Genomic DNA extraction

Total genomic DNA was extracted from mucosal plugs using the protocol for Gram positive bacteria of the DNeasy Blood and Tissue Kit (Qiagen, Inc., Toronto, ON), and from feces and digesta samples (200 ± 5 mg) using the QIAamp DNA Stool Mini Kit (Qiagen, Inc.). Both protocols were conducted according to the manufacturer’s recommendations. Concentrations of genomic DNA from all samples were verified by electrophoresis in a 1% TAE agarose gel.

### T-RFLP community analysis

DNA encoding the 16S rRNA gene was amplified by PCR [40]. Each reaction consisted of 2 μl of genomic DNA (≈10 ng), 2.0 μl of 1X PCR buffer, 0.1 μl of each deoxynucleoside triphosphate (0.2 mM), 2.0 μl of acetylated bovine serum albumin (BSA; Promega, Madison, WI; 0.1 μg μl^-1^), 0.1 μl of Taq DNA polymerase (Qiagen, Inc.; 5 units μl^-1^), 1.0 μl each of the bacterial primers 27F-labeled with 6-fam and 1492R (0.5 μM) [41], and 11.5 μl Optima water (Fisher Scientific, Ottawa, ON). PCR conditions were 95°C for 15 min; 34 cycles consisting of 94°C for 30 s, 53°C for 90 sec, and 72°C for 1 min; and a final extension period at 72°C for 10 min. All PCR reactions were performed in triplicate, and pooled. All amplicons were electrophoresed in a 1% TAE agarose gel relative to a 100 bp DNA ladder (Promega). A single amplicon of ≈ 1500 base pairs was observed in most samples, and these amplicons were purified using QIAquick PCR purification Kit (Qiagen, Inc.). For some mucosal samples, non-specific amplification was observed, and in such cases, the target amplicon was recovered from 1% TAE agarose using QIAquick Gel Extraction Kit (Qiagen, Inc.). DNA concentrations were quantified using a TD 360 Mini Fluorometer (Turner Designs, Sunnyvale, CA) using TNE buffer / Hoescht dye. If required, DNA concentrations were also quantified by agarose gel electrophoresis. Concentrations of DNA in all samples were standardized to 25 ng μl^-1^ using Optima water.

Restriction digests were carried out in duplicate in a mixture containing 75 ng of the purified PCR product, 3 units of HaeIII (Invitrogen Canada Inc., Burlington, ON), 2.5 μl of enzyme buffer, and Optima water to a final volume of 25 μl. Samples were incubated at 37°C for 2 h in the dark, and ethanol precipitation was performed to stop the reaction by adding 50 μl of 95% ethanol and 2 μl of sodium acetate (pH 5.2) to each sample. Samples were incubated for 20 min at 20°C, and centrifuged for 20 min (13,200 × g) to pellet DNA. Nucleic acids were washed by adding 500 μl of 70% ethanol, followed by centrifugation at 13,200 × g for 5 min.

After ethanol precipitation, samples were air dried overnight in the dark, re-suspended in 9.25 μl of Hi Di formamide (Applied Biosystems Canada, Streetsville, ON) and 0.25 μl of LIZ600 size standard marker (Applied Biosystems Canada, Streetsville, ON), denatured at 95°C for 3 min, and immediately placed on ice. Fluorescent labeled terminal restriction fragments (T-RFs) were separated in POP7 polymer using a 3130 Genetic Analyzer (Applied Biosystems Canada), and analyses were performed on T-RFs ranging in size from 50 to 580 base pairs covering V1 to V3 of the 16S rRNA gene. Two electropherograms were obtained per sample (i.e. separate runs), and each electropherogram was analyzed separately using GeneMapper software version 4.0 with the Local Southern size calling method (Applied Biosystems Canada).

A tabulated raw data file from GeneMapper was uploaded to T-RFLP Analysis Expedited (T-REX) [42]. Using T-RF peak height, ‘true’ peaks were distinguished from background fluctuations in fluorescence using a standard deviation of three [43]. Only T-RFs that were common to both electropherograms were used in analyses. T-RFs were then aligned using a clustering method [44] and a clustering threshold of 0.5 base pairs (bp). Two separate data matrices were constructed for downstream analyses; one matrix for T-RF presence/absence and one matrix using T-RF relative abundance data. The data matrices were imported to Bionumerics software version 5.1 (Applied Maths Inc., Austin, TX) for cluster and genetic similarity analyses [40]. Cluster analysis was performed on the T-RF presence/absence data using the Dice coefficient and on T-RF relative abundance data using Bray Curtis. The statistical significance of each group was tested by grouping the treatment replicates within each intestinal location, and comparing the within and between group similarities with randomization tests using 1000 iterations (Applied Maths Inc.) [40]. A probability level of ≤0.05 was used to distinguish distinct groups in a two way comparison (i.e. group A compared to group B and visa-versa). To further explore similarities or dissimilarities in data, non-metric multi-dimensional scaling (NMS) was applied using SAS (SAS Institute Inc., Cary, NC) and three dimensional NMS plots were graphed using SigmaPlot (Systat Software Inc., Chicago, IL). To determine the number of shared and unique OTUs, Venn diagrams were constructed using the T-RF presence/absence data matrix from T-REX. Analysis of variance (ANOVA) for occurrence of T-RFs was performed as a completely randomized design, using the MIXED procedure of SAS (SAS Institute Inc.), with location, treatment, and their interaction included in the model as fixed effects. The repeated measurement statement was applied given sample locations were correlated within animal, and the proper error structure was determined using Akaike’s information criterion and the Bayesian information criterion. Least squares were generated for significant effects and Fisher's protected least significant difference test was used to compare differences among means of interest.

### Clone library community analysis

Clone libraries were constructed from control treatment cattle given that the analysis of T-RFLP fingerprints indicated that communities in the intestines of cattle administered AS700 did not differ appreciably from those in control treatment cattle. Clone libraries were constructed in duplicate (i.e. to minimize potential PCR bias) from DNA isolated from mucosa from the central jejunum, mucosa from the ileum, mucosa from the cecum, mucosa from the descending colon, and digesta from the descending colon. As T-RFLP analyses indicated that community fingerprints were relatively consistent across replicate animals, DNA from all five animals was combined in equal concentrations into a composite mixture and collective libraries were constructed and analyzed.

DNA encoding the 16S rRNA gene was amplified by PCR using bacterial primers as described above except that unlabeled 27F was used. PCR conditions were: 95°C for 15 min; 25 (digesta), 30 (mucosa from the ileum, cecum and descending colon), or 35 (mucosa from the central jejunum) cycles consisting of 94°C for 30 sec, 53°C for 90 sec, and 72°C for 1 min; and a final extension period at 72°C for 10 min. In an attempt to minimize PCR bias, the fewest PCR cycles possible were used.

Traditional clone libraries were constructed using the pGEM® T Easy Vector system with JM109 competent cells (Promega). For ligation, 3 μl of the PCR product and the manufacturers’ reagents for a "standard reaction" were incubated overnight at 4°C. The entire ligation product was then added to 100 μl of the competent cells which were transformed chemically following instructions of the manufacturer with the exception that β-mercaptoethanol was not used. The transformation mixture was plated on 200 ml Luria Burtani (LB) agar containing 10 mg ml^-1^ filter sterilized ampicillin (amp). To induce color differentiation, 320 μl X-Gal in dimethylformamide (50 mg ml^-1^) and 100 μl of 10 mM Isopropyl β-D-1 thiogalactopyranoside were added to the agar. *Escherichia coli* cultures were incubated overnight at 37°C.

Colonies potentially containing a 16S rRNA insert (i.e. white colonies) were picked using a QPIX robot (Genetix Ltd, San Jose, CA) and prepared for Sanger sequencing. Picked colonies were first grown overnight at 37°C in 150 μl of LB amp (100 μg ml^-1^) freezing medium and colony PCR was subsequently performed to ascertain the presence of an insert. Each reaction consisted of 1 μl of *E*. *coli* cells, 2.0 μl of 1X PCR buffer, 0.1 μl of each deoxynucleoside triphosphate (0.2 mM), 2.0 μl of BSA (0.1 μg μl^-1^), 0.1 μl of Taq DNA polymerase (5 units μl^-1^; Qiagen, Inc.), 1.0 μl each of M13 Forward and M13 Reverse primers, and 12.5 μl of Optima water. PCR conditions were: 95°C for 15 min; 35 cycles at 95°C for 30 sec, 54°C for 45 sec, and 72°C for 90 sec; and a final extension period at 72°C for 6 min. To ensure that the PCR product contained 16S rDNA, gel electrophoresis (1% TAE agarose) was performed.

Sanger sequencing was performed on 192 16S rDNA clones each from the central jejunal and ileal mucosa, 384 clones each from the cecal and descending colonic mucosa, and 384 clones from digesta within the descending colon. The PCR product was purified using the MiniElute 96 UF PCR Purification Kit (Qiagen, Inc.). A wash step was performed to increase purity of the DNA, and the recovery of the eluate was completed with 30 μl of Optima water. Sanger sequencing was conducted by Macrogen Corporation (Rockville, MD) using an ABI3730XL machine (Applied Biosystems, Foster City, CA) and the primers 27F and 1492R [41].

Geneious (Biomatters Ltd, Auckland, New Zealand) was used to assemble contigs, and examine electropherograms to ensure proper base calling. Low quality sequences were excluded from further analyses. Sequences were aligned with the GreenGenes NAST alignment tool [45] and putative chimeras were detected using Mallard [46] and the GreenGenes chimera check [47]. Putative chimeras were confirmed with Pintail [48], and were excluded from subsequent analyses. S-Libshuff web [49] was used to ensure that there was not a significant difference between replicate libraries (P > 0.05). Replicate libraries were combined, and Mothur was used to construct rarefaction curves, define OTUs, and estimate community richness (i.e. Chao1 and ACE estimators) and community diversity (i.e. Shannon, non-parametric Shannon and Simpson indices) for each library [50]. Near full length 16S rRNA clone sequences (831 clones in total), and reference sequences and cloned sequences for each library were aligned using the GreenGenes NAST alignment [45], and trees were subsequently constructed in Geneious (Biomatters Ltd); reference sequences (i.e. best type strain match) were obtained from the SEQ Match function of the Ribosomal Database Project (RDP; http://rdp.cme.msu.edu/). Sequence data for the manual construction of a non-linear heat map and taxon frequency pie graphs were obtained using RDP Classifier. The sequence data were accessioned in the GenBank database under the numbers: JX095670 to JX095848 for the middle jejunum (mucosa-associated); JX095849 to JX096028 for the ileum (mucosa-associated); JX094956 to JX095303 for the cecum (mucosa-associated); JX096029 to JX096391 for the descending colon (mucosa-associated); and JX095304 to JX095669 for the descending colon (digesta).

### Quantitative PCR

To quantify total bacteria, genomic DNA extracted from mucosa and digesta was subjected qPCR for total bacteria using the primers, HDA1 and HDA2 [51]; these primers were evaluated by SPYDER *in silico* and were found to detect ≈ 88% of bacterial 16S rDNA sequences within the RDP database [52]. Each reaction consisted of 10 μl of 2X QuantiTect SYBR Green (Qiagen, Inc.), 1.8 μl of HDA1 (10 μM), 1.2 μl of HDA2 (10 μM), 2.0 μl of BSA (1 mg ml^-1^), 3 μl of nuclease free water (Qiagen, Inc.), and 2 μl of template. Samples were amplified and fluorescence was detected on an Mx3005P thermocycler (Agilent Technologies Inc.). Thermocycler conditions consisted of an activation cycle of 95°C for 15 min, followed by 40 cycles at 94°C for 15 sec, 56°C for 30 sec, and 72°C for 30 sec; qPCR reaction conditions were optimized with DNA from *E*. *coli* (ATCC 25922). Copy numbers of the 16S rRNA gene in extracted genomic DNA were interpolated from a standard curve of genomic DNA from *E*. *coli* (ATCC 25922) with 1 ng of *E*. *coli* DNA containing 1.4 × 10^6^ genome copies [53].

For analysis of variance, copy number was log_10_ transformed to normalize variance and data were analyzed using the mixed procedure of SAS (SAS Institute Inc, Cary NC). Treatment and time or location and their respective interactions were included in the model as fixed effects. The repeated measurement statement was applied given that mucosal and digesta samples collected at different locations were correlated within animal. The appropriate error structure was determined using Akaike’s information criterion and the Bayesian information criterion, and the univariate procedure (SAS Institute, Inc.) was used to produce normal probability plots to confirm normality. Least squares were generated for significant effects and Fisher's protected least significant difference test was used to compare differences between treatments and locations.

## Competing interests

The authors declare that they have no competing interests.

## Authors’ contributions

KLR carried out the T-RFLP and clone library analyses, and co-drafted the manuscript. MCT assisted with statistical analyses. LJY and LBS participated in the design of the study. GDI conceived the study, participated in the design of the study, collected intestinal samples, prepared figures, and co-drafted the manuscript. All authors have approved the manuscript.

## Supplementary Material

Additional file 1: Figure S1 Unrooted phylogenetic tree of mucosa-associated bacteria within the central jejunum of beef cattle not administered antimicrobials (179 clones) and closest reference bacteria (NCBI Accession Number in parentheses) where: 1 = *Oscillibacter valericigenes* (AB238598); 2 = *Acetivibrio cellulolyticus* (L35516); 3 = *Clostridium colicanis* (AJ420008); 4 = *Clostridium disporicum* (Y18176); 5 = *Clostridium irregulare* (X73447); 6 = *Lactobacillus amylovorus* (AY944408); 7 = *Lactobacillus mucosae* (AF126738); 8 = *Syntrophococcus sucromutans* (AF202264); 9 = *Phascolarctobacterium faecium* (X72865); 10 = *Mycobacterium aubagnense* (AY859683); 11 = *Propionibacterium acnes* (AB042288); 12 = *Knoellia aerolata* (EF553529); 13 = *Bifidobacterium saeculare* (D89328); 14 = *Campylobacter jejuni* (DQ174144); 15 = *Campylobacter coli* (AF372092); 16 = *Helicobacter canadensis* (AF262037); 17 = *Sediminibacterium salmoneum* (EF407879); 18 = *Ferruginibacter lapsinanis* (FJ177532); 19 = *Ferruginibacter alkalilentus* (FJ177530); 20 = *Prevotella copri* (AB064923); 21 = *Sorangium cellulosum* (EU240497); 22 = *Desulfuromonas acetexigens* (U23140); 23 = *Steroidobacter denitrificans* (EF605262); 24 = *Nevskia soli* (EF178286); 25 = *Ralstonia pickettii* (AY741342); 26 = *Ralstonia insidiosa* (AF488779); 27 = *Janthinobacterium lividum* (Y08846); 28 = *Delftia tsuruhatensis* (AB075017); 29 = *Shigella flexneri* (X96963); 30 = *Enterobacter asburiae* (AB004744); 31 = *Magnetospirillum magnetotacticum* (Y10110); 32 = *Hyphomicrobium facile* (Y14309); 33 = *Mesorhizobium pluifarium* (Y14158); 34 = *Caulobacter segnis* (AB023427); 35 = *Caulobacter henriccii* (AJ227758); 36 = *Caulobacter mirabilis* (AJ227774); 37 = *Phenylobacterium lituiforme* (AY534887); 38 = *Phenylobacterium immobile* (Y18216); 39 = *Bradyrhizobium yuanmingense* (AF193818); 40 = *Bradyrhizobium pachyrhizi* (AY624135); 41 = *Bradyrhizobium betae* (AY372184); and 42 = *Bradyrhizobium liaoningense* (AF208513). Click here for file

Additional file 2: Figure S2 Unrooted phylogenetic tree of mucosa-associated bacteria within the ileum of beef cattle not administered antimicrobials (181 clones) and closest reference bacteria (NCBI Accession Number in parentheses) where: 1 = *Clostridium irregulare* (X73447); 2 = *Thermotalea metallivorans* (EU443727); 3 = *Anaerovorax odorimutans* (AJ251215); 4 = *Mogibacterium vescum* (AB021702); 5 = *Bulleidia extructa* (AF220064); 6 = *Clostridium celatum* (X77844); 7 = *Clostridium disporicum* (Y18176); 8 = *Clostridium colicanis* (AJ420008); 9 = *Desulfitobacterium metallireducens* (AF297871); 10 = *Anaerovibrio lipolyticus* (AB034191); 11 = *Turicibacter sanguinis* (AF349724); 12 = *Lactobacillus curvatus* (AM113777); 13 = *Lactobacillus mucosae* (AF126738); 14 = *Lactobacillus amylovorus* (AY944408); 15 = *Lactobacillus ruminis* (AB326354); 16 = *Hydrogenoanaerobacterium saccharovorans* (EU158190); 17 = *Oscillibacter valericigenes* (AB238598); 18 = *Eubacterium plautii* (AY724678); 19 = *Papillibacter cinnamivorans* (AF167711); 20 = *Bifidobacterium saeculare* (D89328); 21 = *Barnesiella intestinihominis* (AB370251); 22 = *Shigella flexneri* (X96963); 23 = *Escherichia fergusonii* (AF530475); 24 = *Campylobacter jejuni* (DQ174144); 25 = *Campylobacter curvus* (DQ174165); 26 = *Clostridium sufflavum* (AB267266); 27 = *Acetivibrio celluloyticus* (L35516); 28 = *Clostridium clariflavum* (AB186359); 29 = *Syntrophococcus sucromutans* (AF202264); 30 = *Blautia wexlerae* (EF036467); 31 = *Blautia luti* (AJ133124); 32 = *Blautia hydrogenotrophica* (X95624); 33 = *Coprococcus comes* (EF031542); 34 = *Hespellia stercorisuis* (AF445264); 35 = *Coprococcus catus* (AB038359); 36 = *Roseburia inulinivorans* (AJ270473); and 37 = *Clostridium aldenense* (DQ279736). Click here for file

Additional file 3: Figure S3 Unrooted phylogenetic tree of mucosa-associated bacteria within the cecum of beef cattle not administered antimicrobials (349 clones) and closest reference bacteria (NCBI Accession Number in parentheses) where: 1 = *Parabacteroides goldsteinii* (AY974070); 2 = *Barnesiella viscericola* (AB267809); 3 = *Barnesiella intestinihominis* (AB370251); 4 = *Paraprevotella clara* (AB331896); 5 = *Bacteroides plebeius* (AB200217); 6 = *Bacteroides coprocola* (AB200224); 7 = *Bacteroides massiliensis* (AY126616); 8 = *Prevotella histicola* (EU126661); 9 = *Alistipes onderdonkii* (AY974071); 10 = *Alistipes finegoldii* (AY643083); 11 = *Rikenella microfusus* (L16498); 12 = *Pedobacter hartonius* (AM491371); 13 = *Pedobacter cryoconitis* (AJ438170); 14 = *Sediminibacterium salmoneum* (EF407879); 15 = *Treponema porcinum* (AY518274); 16 = *Victivallis vadensis* (AY049713); 17 = *Akkermansia muciniphila* (AY271254); 18 = *Planktothricoides raciborskii* (AB045960); 19 = *Acidobacterium capsulatum* (CP001472); 20 = *Steroidobacter denitrificans* (EF605262); 21 = *Aeromonas salmonicida* (X60407); 22 = *Aeromonas jandaei* (X60413); 23 = *Ruminobacter amylophilus* (Y15992); 24 = *Pseudolabrys taiwanensis* (DQ062742); 25 = *Caulobacter henricii* (AJ227758); 26 = *Clostridium irregulare* (X73447); 27 = *Thermotalea metallivorans* (EU443727); 28 = *Anaerovorax odorimutans* (AJ251215); 29 = *Eubacterium infirmum* (U13039); 30 = *Clostridium disporicum* (Y18176); 31 = *Clostridium chartatabidum* (X71850); 32 = *Lactobacillus mucosae* (AF126738); 33 = *Lactobacillus amylovorus* (AY944408); 34 = *Lactobacillus ruminis* (AB326354); 35 = *Bacillus humi* (AJ627210); 36 = *Eubacterium callanderi* (X96961); 37 = *Desulfosporosinus lacus* (AJ582757); 38 = *Desulfitobacterium metallireducens* (AF297871); 39 = *Desulfitibacter alkalitolerans* (AY538171); 40 = *Clostridium clariflavum* (AB186359); 41 = *Acetivibrio cellulolyticus* (L35516); 42 = *Clostridium sufflavum* (AB267266); 43 = *Clostridium caenicola* (AB221372); 44 = *Ethanoligenens harbinense* (AY295777); 45 = *Clostridium cellulosi* (L09177); 46 = *Acetanaerobacterium elongatum* (AY487928); 47 = *Hydrogenoaerobacterium saccharovorans* (EU158190); 48 = *Anaerotruncus colihominis* (AJ315980); 49 = *Ruminococcus albus* (L76598); 50 = *Ruminococcus bromii* (L76600); 51 = *Faecalibacterium prausnitzii* (AJ41395); 52 = *Butyricicoccus pullicaecorum* (EU410376); 53 = *Oscillibacter valericigenes* (AB238598); 54 = *Eubacterium plautii* (AY724678); 55 = *Papillibacter cinnamivorans* (AF167711); 56 = *Lutispora thermophila* (AB186360); 57 = *Gracilibacter thermotolerans* (DQ117465); 58 = *Clostridium jejuense* (AY494606); 59 = *Anaerosporobacter mobilis* (AY534872); 60 = *Robinsoniella peoriensis* (AF445285); 61 = *Hespellia porcina* (AF445239); 62 = *Ruminococcus lactaris* (L76602); 63 = *Coprococcus comes* (EF031542); 64 = *Coprococcus eutactus* (EF031543); 65 = *Blautia schinkii* (X94965); 66 = *Blautia luti* (AJ133124); 67 = *Blautia hydrogenotrophica* (X95624); 68 = *Roseburia intestinalis* (AJ312385); 69 = *Roseburia faecis* (AY305310); 70 = *Coprococcus catus* (AB038359); 71 = *Clostridium lavalense* (EF564277); 72 = Clostridium aldenense (DQ279736); 73 = *Syntrophococcus sucromutans* (AF202264); 74 = *Pseudobutryrivibrio ruminis* (X95893); 75 = *Parasporobacterium paucivorans* (AJ272036); 76 = *Megasphaera micronuciformis* (AF473834); 77 = *Phascolarctobacterium faecium* (X72865); and 78 = *Anaerovibrio lipolyticus* (AB034191). Click here for file

Additional file 4: Figure S4 Unrooted phylogenetic tree of mucosa-associated bacteria within the descending colon of beef cattle not administered antimicrobials (368 clones) and closest reference bacteria (NCBI Accession Number in parentheses) where: 1 = *Oscillibacter valericigenes* (AB238598); 2 = *Eubacterium plautii* (AY724678); 3 = *Sporobacter termitidis* (Z49863); 4 = *Papillibacter cinnamivorans* (AF167711); 5 = *Butyricicoccus pullicaecorum* (EU410376); 6 = *Ethanoligenens harbinense* (AY295777); 7 = *Clostridium cellulosi* (L09177); 8 = *Acetanaerobacterium elongatum* (AY487928); 9 = *Hydrogenoanaerobacterium saccharovorans* (EU158190); 10 = *Anaerotruncus colihominis* (AJ315980); 11 = *Ruminococcus albus* (L76598); 12 = *Ruminococcus flavefaciens* (L76603); 13 = *Clostridium sporosphaeroides* (X66002); 14 = *Ruminococcus bromii* (L76600); 15 = *Clostridium caenicola* (AB221372); 16 = *Clostridium clariflavum* (AB186359); 17 = *Acetivibrio cellulolyticus* (L35516); 18 = *Macrococcus brunensis* (AY119686); 19 = *Bacillus funiculus* (AB049195); 20 = *Vulcanibacillus modesticaldus* (AM050346); 21 = *Lactobacillus amylovorus* (AY944408); 22 = *Eubacterium tortuosum* (L34683); 23 = *Erysipelothrix inopinata* (AJ550617); 24 = *Turicibacter sanguinis* (AF349724); 25 = *Acholeplasma axanthum* (AF412968); 26 = *Desulfosporosinus lacus* (AJ582757); 27 = *Desulfitobacterium metallireducens* (AF297871); 28 = *Thermincola ferriacetica* (AY631277); 29 = *Anaerovibrio lipolyticus* (AB034191); 30 = *Phascolarctobacterium faecium* (X72865); 31 = *Dietzia maris* (X79290); 32 = *Rubrobacter taiwanensis* (AF465803); 33 = *Akkermansia muciniphila* (AY271254); 34 = *Rhodopirellula baltica* (BX294149); 35 = *Tistrella mobilis* (AB071665); 36 = *Roseburia intestinalis* (AJ312385); 37 = *Roseburia faecis* (AY305310); 38 = *Roseburia inulinivorans* (AJ270473); 39 = *Lachnobacterium bovis* (AF298663); 40 = *Blautia luti* (AJ133124); 41 = *Blautia hydrogenotrophica* (X95624); 42 = *Coprococcus catus* (AB038359); 43 = *Anaerosporobacter mobilis* (AY534872); 44 = *Ruminococcus lactaris* (L76602); 45 = *Ruminococcus gauvreauii* (EF529620); 46 = *Robinsoniella peoriensis* (AF445285); 47 = *Hespellia porcina* (AF445239); 48 = *Dorea longicatena* (AJ132842); 49 = *Coprococcus eutactus* (EF031543); 50 = *Coprococcus comes* (EF031542); 51 = *Clostridium jejuense* (AY494606); 52 = *Catonella morbi* (X87151); 53 = *Clostridium irregulare* (X73447); 54 = *Eubacterium sulci* (AJ006963); 55 = *Anaerovorax odorimutans* (AJ251215); 56 = *Thermotalea metallivorans* (EU443727); 57 = *Soehngenia saccharolytica* (AY353956); 58 = *Clostridium disporicum* (Y18176); 59 = *Clostridium butyricum* (AJ458420); 60 = *Clostridium puniceum* (X71857); 61 = *Paraprevotella xylaniphila* (AB331897); 62 = *Paraprevotella clara* (AB331896); 63 = *Prevotella salivae* (AB108826); 64 = *Prevotella nanceiensis* (AY957555); 65 = *Bacteroides plebeius* (AB200217); 66 = *Bacteroides coprocola* (AB200224); 67 = *Bacteroides massiliensis* (AY126616); 68 = *Bacteroides gallinarum* (AB253732); 69 = *Paludibacter propionicigenes* (AB078842); 70 = *Petrimonas sulfuriphila* (AY570690); 71 = *Barnesiella viscericola* (AB267809); 72 = *Barnesiella intestinihominis* (AB370251); 73 = *Alistipes shahii* (AY974072); 74 = *Alistipes finegoldii* (AY643083); 75 = *Alistipes onderdonkii* (AY974071); 76 = *Rikenella microfusus* (L16498); 77 = Pedobacter cryoconitis (AJ438170); 78 = *Lutispora thermophila* (AB186360); 79 = *Gracilibacter thermotolerans* (DQ117465); and 80 = *Natronovigra wadinatrunensis* (EU338489). Click here for file

Additional file 5: Figure S5 Unrooted phylogenetic tree of bacteria in digesta within the descending colon of beef cattle not administered antimicrobials (367 clones) and closest reference bacteria (NCBI Accession Number in parentheses) where: 1 = *Alistipes onderdonkii* (AY974071); 2 = *Alistipes finegoldii* (AY643083); 3 = *Rikenella microfusus* (L16498); 4 = *Bacteroides plebeius* (AB200217); 5 = *Bacteroides coprocola* (AB200224); 6 = *Bacteroides massiliensis* (AY126616); 7 = *Bacteroides gallinarum* (AB253732); 8 = *Paraprevotella xylaniphila* (AB331897); 9 = *Paraprevotella clara* (AB331896); 10 = *Prevotella copri* (AB064923); 11 = *Prevotella nanceiensis* (AY957555); 12 = *Barnesiella viscericola* (AB267809); 13 = *Barnesiella intestinihominis* (AB370251); 14 = *Parabacteroides merdae* (AB238928); 15 = *Paludibacter propionicigenes* (AB078842); 16 = *Pedobacter cryoconitis* (AJ438170); 17 = *Phascolarctobacterium faecium* (X72865); 18 = *Propionispira arboris* (Y18190); 19 = *Lutispora thermophila* (AB186360); 20 = *Gracilibacter thermotolerans* (DQ117465); 21 = *Natronovirga wadinatrunensis* (EU338489); 22 = *Caloramator australicus* (EU409943); 23 = *Clostridium clariflavum* (AB186359); 24 = *Acetivibrio cellulolyticus* (L35516); 25 = *Sporobacter termitidis* (Z49863); 26 = *Papillibacter cinnamivorans* (AF167711); 27 = *Eubacterium plautii* (AY724678); 28 = *Oscillibacter valericigenes* (AB238598); 29 = *Butyricicoccus pullicaecorum* (EU410376); 30 = *Clostridium sporospaeroides* (X66002); 31 = *Ruminococcus bromii* (L76600); 32 = *Ethanoligenens harbinense* (AY295777); 33 = *Clostridium cellulosi* (L09177); 34 = *Acetanaerobacterium elongatum* (AY487928); 35 = *Hydrogenoanaerobacterium saccharovorans* (EU158190); 36 = *Anaerotruncus colihominis* (AJ315980); 37 = *Ruminococcus albus* (L76598); 38 = *Ruminococcus flavefaciens* (L76603); 39 = *Desulfonispora thiosulfatigenes* (Y18214); 40 = *Desulfitobacterium metallireducens* (AF297871); 41 = *Desulfitobacterium chloroespirans* (U68528); 42 = *Desulfosporosinus auripigmenti* (AJ493051); 43 = *Clostridium irregulare* (X73447); 44 = *Thermotalea metallivorans* (EU443727); 45 = *Eubacterium sulci* (AJ006963); 46 = *Anaerovorax odorimutans* (AJ251215); 47 = *Clostridium saccharoperbutylacetonicum* (U16122); 48 = *Treponema porcinum* (AY518274); 49 = *Succinivibrio dextrinosolvens* (Y17600); 50 = *Acholeplasma axanthum* (AF412968); 51 = *Ruminococcus gauvreauii* (EF529620); 52 = *Robinsoniella peroriensis* (AF445285); 53 = *Clostridium citroniae* (DQ279737); 54 = *Roseburia intestinalis* (AJ312385); 55 = *Roseburia faecis* (AY305310); 56 = *Lachnobacterium bovis* (AF298663); 57 = *Pseudobutyrivibrio ruminis* (X95893); 58 = *Coprococcus comes* (EF031542); 59 = *Ruminococcus lactaris* (L76602); 60 = *Dorea longicatena* (AJ132842); 61 = *Clostridium phytofermentans* (CP000885); 62 = *Anaerosporobacter mobilis* (AY534872); 63 = *Hyphomicrobium zavarzinii* (Y14305); 64 = *Prochlorococcus marinus* (AE017126); 65 = *Planktothricoides raciborskii* (AB045960); and 66 = *Akkermansia muciniphila* (AY271254). Click here for file
