# Supplementary figures and images for: Effect of antimicrobial growth promoter administration on the intestinal microbiota of beef cattle
Source: Gut Pathog. 2013 Apr 11;5:8. doi: 10.1186/1757-4749-5-8 (PMC3639104; doi:10.1186/1757-4749-5-8)

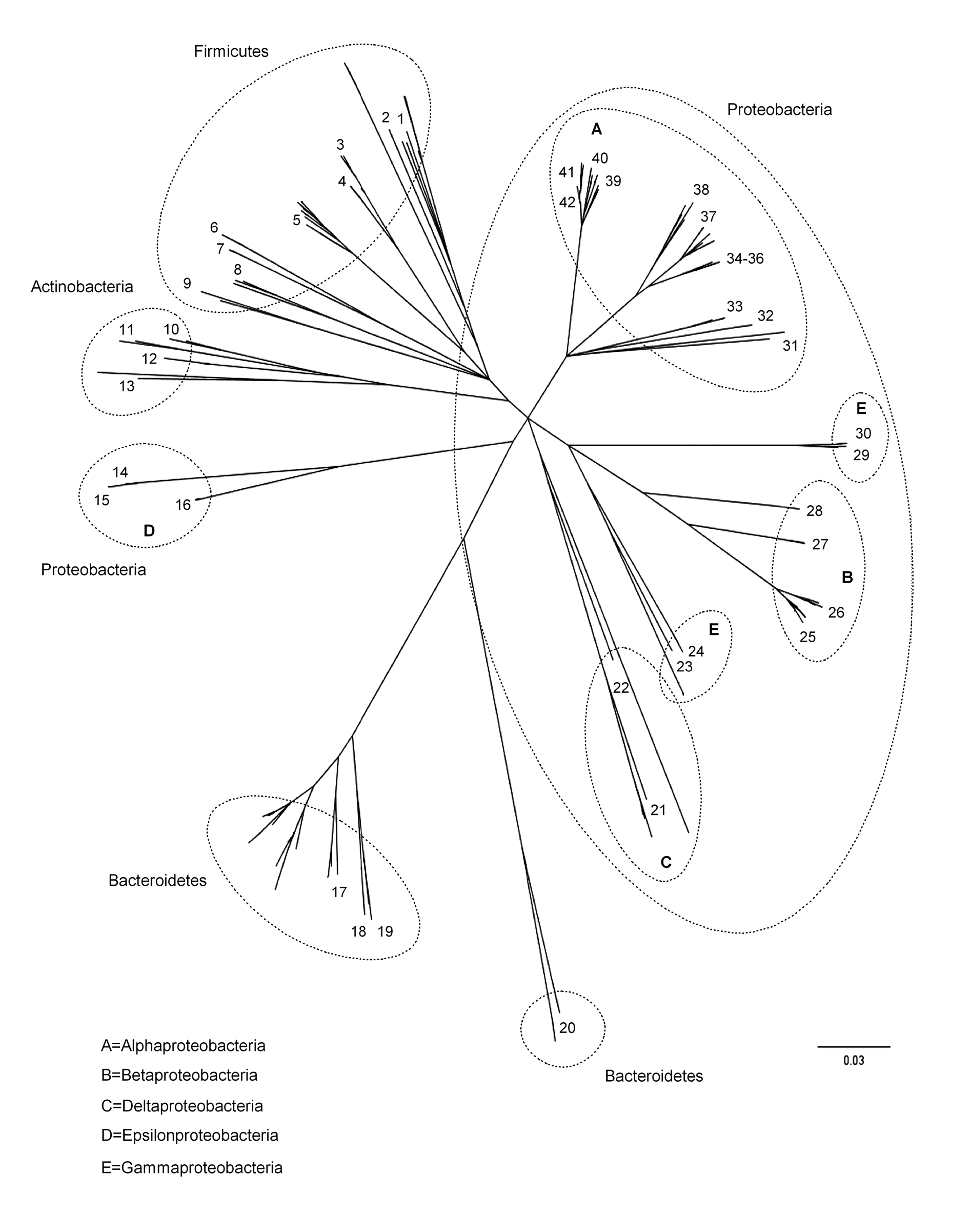

Supplement: Additional file 1: Figure S1 — Unrooted phylogenetic tree of mucosa-associated bacteria within the central jejunum of beef cattle not administered antimicrobials (179 clones) and closest reference bacteria (NCBI Accession Number in parentheses) where: 1 = Oscillibacter valericigenes (AB238598); 2 = Acetivibrio cellulolyticus (L35516); 3 = Clostridium colicanis (AJ420008); 4 = Clostridium disporicum (Y18176); 5 = Clostridium irregulare (X73447); 6 = Lactobacillus amylovorus (AY944408); 7 = Lactobacillus mucosae (AF126738); 8 = Syntrophococcus sucromutans (AF202264); 9 = Phascolarctobacterium faecium (X72865); 10 = Mycobacterium aubagnense (AY859683); 11 = Propionibacterium acnes (AB042288); 12 = Knoellia aerolata (EF553529); 13 = Bifidobacterium saeculare (D89328); 14 = Campylobacter jejuni (DQ174144); 15 = Campylobacter coli (AF372092); 16 = Helicobacter canadensis (AF262037); 17 = Sediminibacterium salmoneum (EF407879); 18 = Ferruginibacter lapsinanis (FJ177532); 19 = Ferruginibacter alkalilentus (FJ177530); 20 = Prevotella copri (AB064923); 21 = Sorangium cellulosum (EU240497); 22 = Desulfuromonas acetexigens (U23140); 23 = Steroidobacter denitrificans (EF605262); 24 = Nevskia soli (EF178286); 25 = Ralstonia pickettii (AY741342); 26 = Ralstonia insidiosa (AF488779); 27 = Janthinobacterium lividum (Y08846); 28 = Delftia tsuruhatensis (AB075017); 29 = Shigella flexneri (X96963); 30 = Enterobacter asburiae (AB004744); 31 = Magnetospirillum magnetotacticum (Y10110); 32 = Hyphomicrobium facile (Y14309); 33 = Mesorhizobium pluifarium (Y14158); 34 = Caulobacter segnis (AB023427); 35 = Caulobacter henriccii (AJ227758); 36 = Caulobacter mirabilis (AJ227774); 37 = Phenylobacterium lituiforme (AY534887); 38 = Phenylobacterium immobile (Y18216); 39 = Bradyrhizobium yuanmingense (AF193818); 40 = Bradyrhizobium pachyrhizi (AY624135); 41 = Bradyrhizobium betae (AY372184); and 42 = Bradyrhizobium liaoningense (AF208513). [file 1757-4749-5-8-S1.tiff]

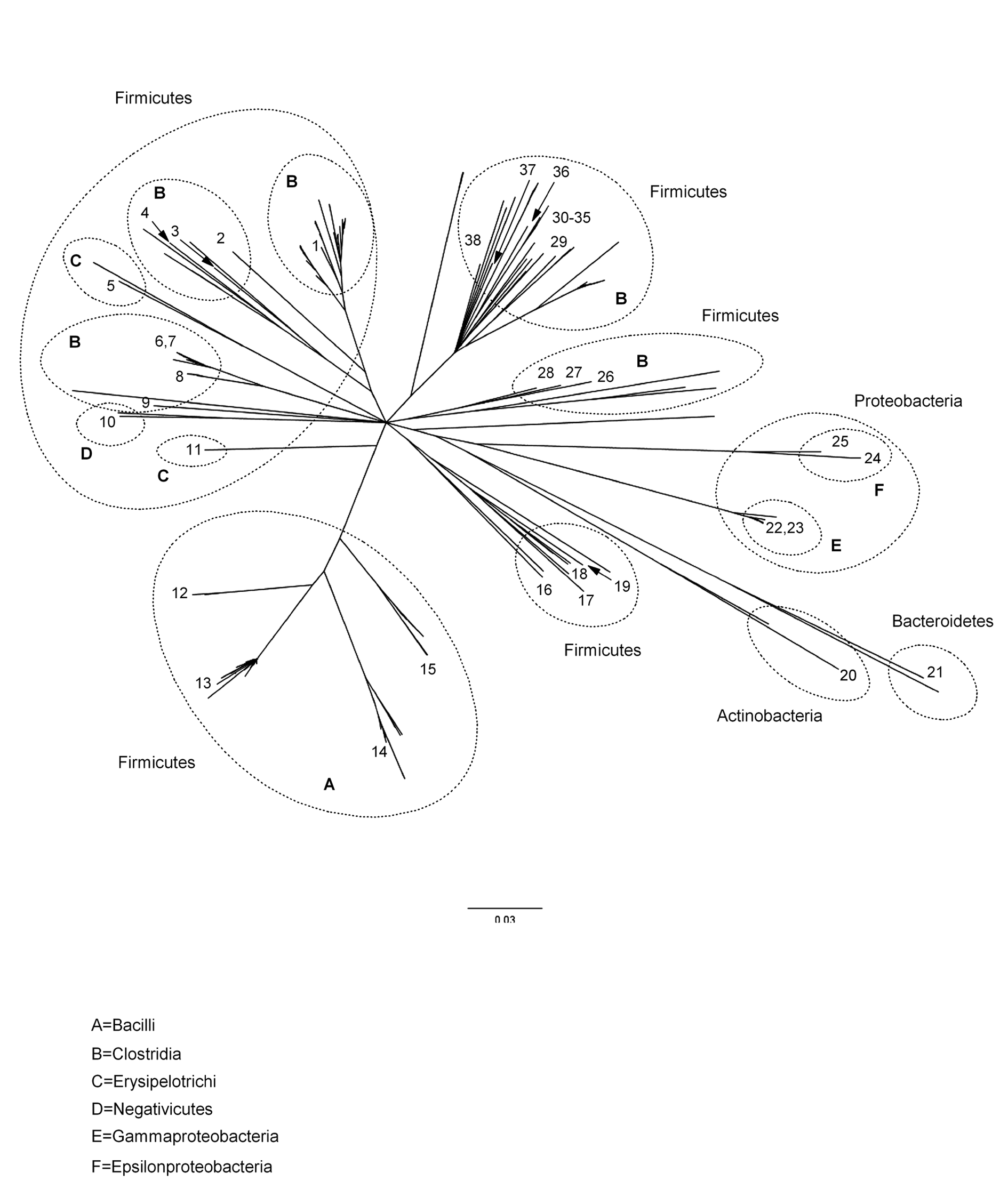

Supplement: Additional file 2: Figure S2 — Unrooted phylogenetic tree of mucosa-associated bacteria within the ileum of beef cattle not administered antimicrobials (181 clones) and closest reference bacteria (NCBI Accession Number in parentheses) where: 1 = Clostridium irregulare (X73447); 2 = Thermotalea metallivorans (EU443727); 3 = Anaerovorax odorimutans (AJ251215); 4 = Mogibacterium vescum (AB021702); 5 = Bulleidia extructa (AF220064); 6 = Clostridium celatum (X77844); 7 = Clostridium disporicum (Y18176); 8 = Clostridium colicanis (AJ420008); 9 = Desulfitobacterium metallireducens (AF297871); 10 = Anaerovibrio lipolyticus (AB034191); 11 = Turicibacter sanguinis (AF349724); 12 = Lactobacillus curvatus (AM113777); 13 = Lactobacillus mucosae (AF126738); 14 = Lactobacillus amylovorus (AY944408); 15 = Lactobacillus ruminis (AB326354); 16 = Hydrogenoanaerobacterium saccharovorans (EU158190); 17 = Oscillibacter valericigenes (AB238598); 18 = Eubacterium plautii (AY724678); 19 = Papillibacter cinnamivorans (AF167711); 20 = Bifidobacterium saeculare (D89328); 21 = Barnesiella intestinihominis (AB370251); 22 = Shigella flexneri (X96963); 23 = Escherichia fergusonii (AF530475); 24 = Campylobacter jejuni (DQ174144); 25 = Campylobacter curvus (DQ174165); 26 = Clostridium sufflavum (AB267266); 27 = Acetivibrio celluloyticus (L35516); 28 = Clostridium clariflavum (AB186359); 29 = Syntrophococcus sucromutans (AF202264); 30 = Blautia wexlerae (EF036467); 31 = Blautia luti (AJ133124); 32 = Blautia hydrogenotrophica (X95624); 33 = Coprococcus comes (EF031542); 34 = Hespellia stercorisuis (AF445264); 35 = Coprococcus catus (AB038359); 36 = Roseburia inulinivorans (AJ270473); and 37 = Clostridium aldenense (DQ279736). [file 1757-4749-5-8-S2.tiff]

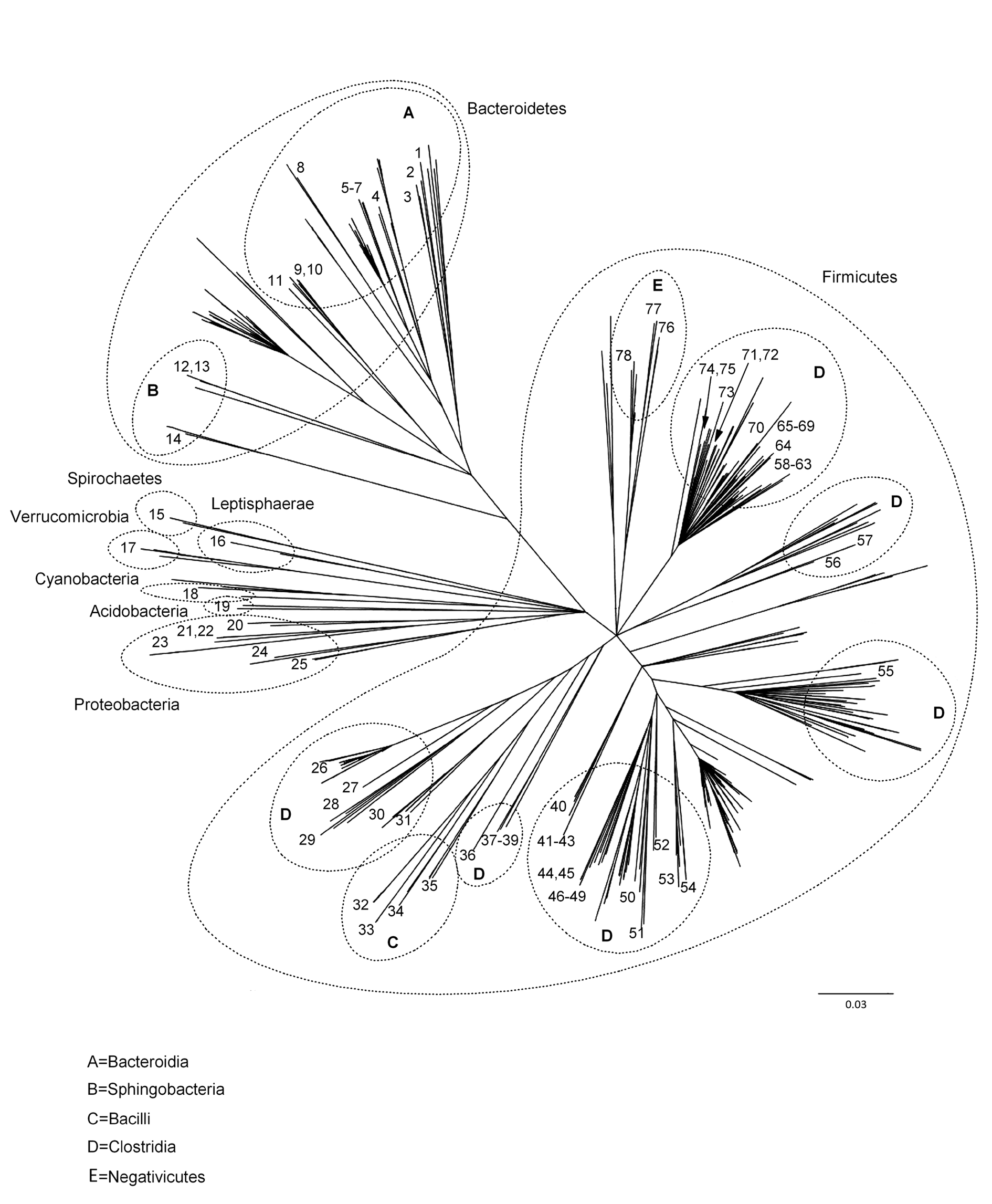

Supplement: Additional file 3: Figure S3 — Unrooted phylogenetic tree of mucosa-associated bacteria within the cecum of beef cattle not administered antimicrobials (349 clones) and closest reference bacteria (NCBI Accession Number in parentheses) where: 1 = Parabacteroides goldsteinii (AY974070); 2 = Barnesiella viscericola (AB267809); 3 = Barnesiella intestinihominis (AB370251); 4 = Paraprevotella clara (AB331896); 5 = Bacteroides plebeius (AB200217); 6 = Bacteroides coprocola (AB200224); 7 = Bacteroides massiliensis (AY126616); 8 = Prevotella histicola (EU126661); 9 = Alistipes onderdonkii (AY974071); 10 = Alistipes finegoldii (AY643083); 11 = Rikenella microfusus (L16498); 12 = Pedobacter hartonius (AM491371); 13 = Pedobacter cryoconitis (AJ438170); 14 = Sediminibacterium salmoneum (EF407879); 15 = Treponema porcinum (AY518274); 16 = Victivallis vadensis (AY049713); 17 = Akkermansia muciniphila (AY271254); 18 = Planktothricoides raciborskii (AB045960); 19 = Acidobacterium capsulatum (CP001472); 20 = Steroidobacter denitrificans (EF605262); 21 = Aeromonas salmonicida (X60407); 22 = Aeromonas jandaei (X60413); 23 = Ruminobacter amylophilus (Y15992); 24 = Pseudolabrys taiwanensis (DQ062742); 25 = Caulobacter henricii (AJ227758); 26 = Clostridium irregulare (X73447); 27 = Thermotalea metallivorans (EU443727); 28 = Anaerovorax odorimutans (AJ251215); 29 = Eubacterium infirmum (U13039); 30 = Clostridium disporicum (Y18176); 31 = Clostridium chartatabidum (X71850); 32 = Lactobacillus mucosae (AF126738); 33 = Lactobacillus amylovorus (AY944408); 34 = Lactobacillus ruminis (AB326354); 35 = Bacillus humi (AJ627210); 36 = Eubacterium callanderi (X96961); 37 = Desulfosporosinus lacus (AJ582757); 38 = Desulfitobacterium metallireducens (AF297871); 39 = Desulfitibacter alkalitolerans (AY538171); 40 = Clostridium clariflavum (AB186359); 41 = Acetivibrio cellulolyticus (L35516); 42 = Clostridium sufflavum (AB267266); 43 = Clostridium caenicola (AB221372); 44 = Ethanoligenens harbinense (AY295777); 45 = Clostridium cellul [file 1757-4749-5-8-S3.tiff]

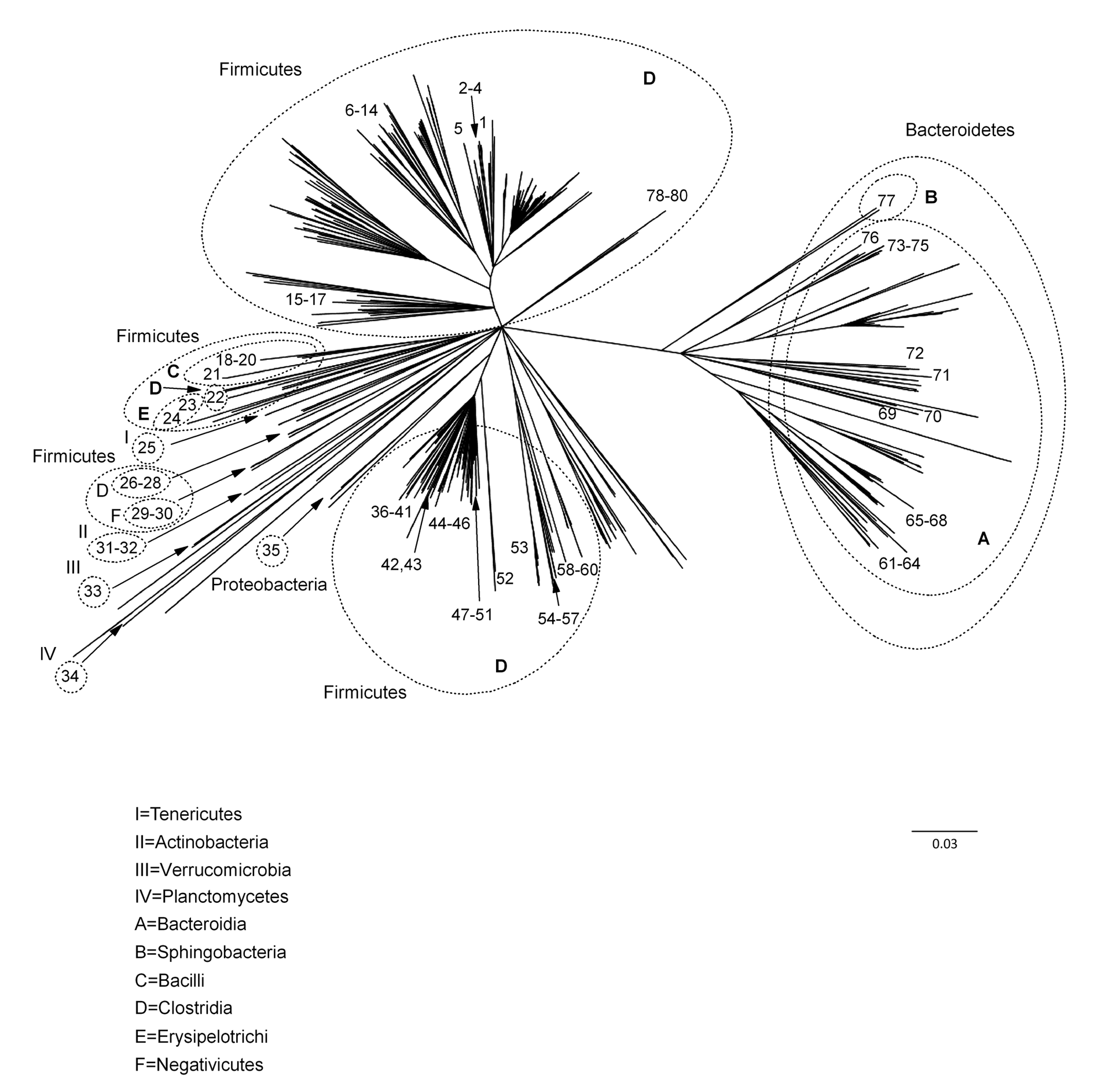

Supplement: Additional file 4: Figure S4 — Unrooted phylogenetic tree of mucosa-associated bacteria within the descending colon of beef cattle not administered antimicrobials (368 clones) and closest reference bacteria (NCBI Accession Number in parentheses) where: 1 = Oscillibacter valericigenes (AB238598); 2 = Eubacterium plautii (AY724678); 3 = Sporobacter termitidis (Z49863); 4 = Papillibacter cinnamivorans (AF167711); 5 = Butyricicoccus pullicaecorum (EU410376); 6 = Ethanoligenens harbinense (AY295777); 7 = Clostridium cellulosi (L09177); 8 = Acetanaerobacterium elongatum (AY487928); 9 = Hydrogenoanaerobacterium saccharovorans (EU158190); 10 = Anaerotruncus colihominis (AJ315980); 11 = Ruminococcus albus (L76598); 12 = Ruminococcus flavefaciens (L76603); 13 = Clostridium sporosphaeroides (X66002); 14 = Ruminococcus bromii (L76600); 15 = Clostridium caenicola (AB221372); 16 = Clostridium clariflavum (AB186359); 17 = Acetivibrio cellulolyticus (L35516); 18 = Macrococcus brunensis (AY119686); 19 = Bacillus funiculus (AB049195); 20 = Vulcanibacillus modesticaldus (AM050346); 21 = Lactobacillus amylovorus (AY944408); 22 = Eubacterium tortuosum (L34683); 23 = Erysipelothrix inopinata (AJ550617); 24 = Turicibacter sanguinis (AF349724); 25 = Acholeplasma axanthum (AF412968); 26 = Desulfosporosinus lacus (AJ582757); 27 = Desulfitobacterium metallireducens (AF297871); 28 = Thermincola ferriacetica (AY631277); 29 = Anaerovibrio lipolyticus (AB034191); 30 = Phascolarctobacterium faecium (X72865); 31 = Dietzia maris (X79290); 32 = Rubrobacter taiwanensis (AF465803); 33 = Akkermansia muciniphila (AY271254); 34 = Rhodopirellula baltica (BX294149); 35 = Tistrella mobilis (AB071665); 36 = Roseburia intestinalis (AJ312385); 37 = Roseburia faecis (AY305310); 38 = Roseburia inulinivorans (AJ270473); 39 = Lachnobacterium bovis (AF298663); 40 = Blautia luti (AJ133124); 41 = Blautia hydrogenotrophica (X95624); 42 = Coprococcus catus (AB038359); 43 = Anaerosporobacter mobilis (AY534872); 44 = Ruminococcus lactaris (L76602); 45 [file 1757-4749-5-8-S4.tiff]

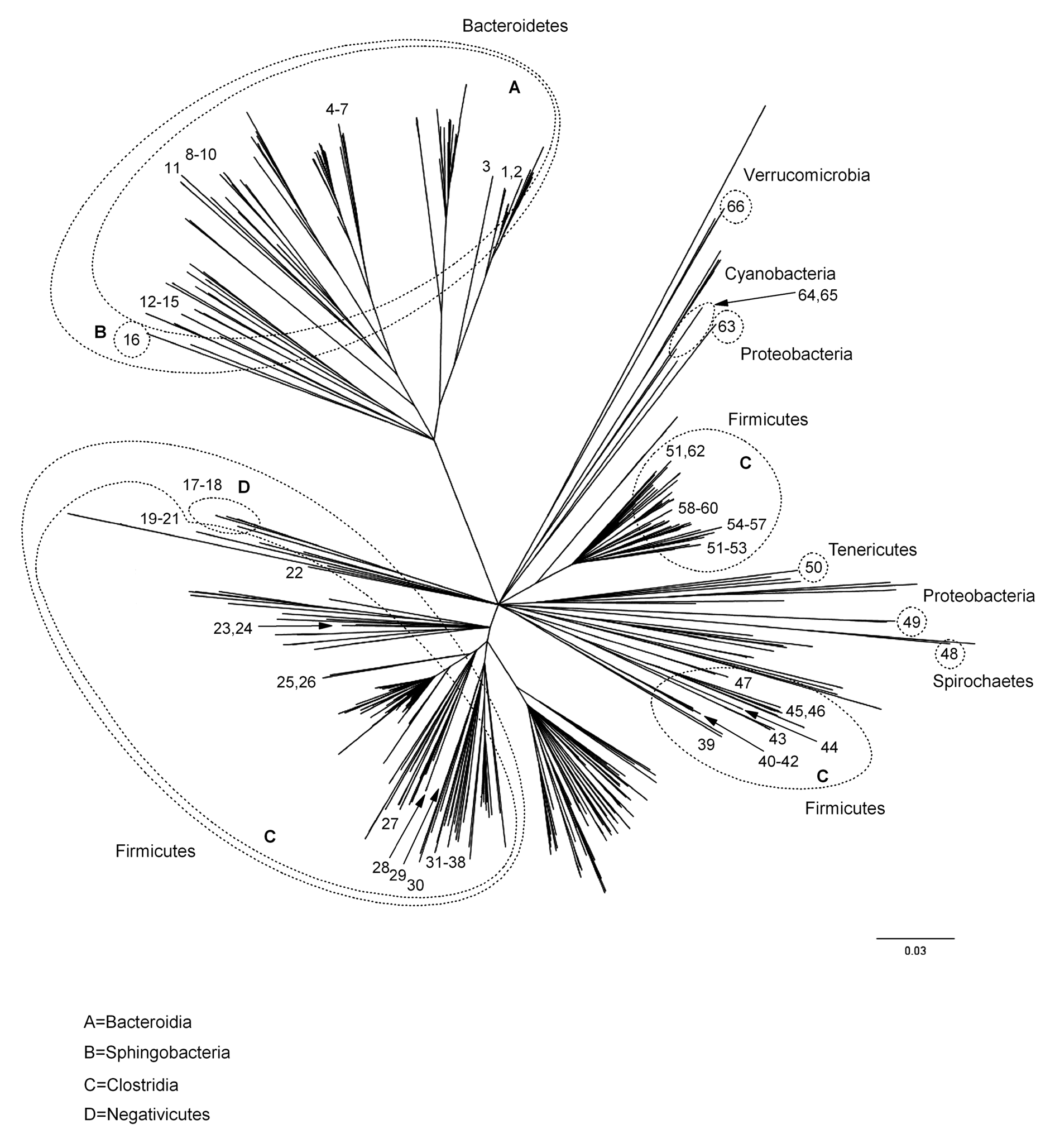

Supplement: Additional file 5: Figure S5 — Unrooted phylogenetic tree of bacteria in digesta within the descending colon of beef cattle not administered antimicrobials (367 clones) and closest reference bacteria (NCBI Accession Number in parentheses) where: 1 = Alistipes onderdonkii (AY974071); 2 = Alistipes finegoldii (AY643083); 3 = Rikenella microfusus (L16498); 4 = Bacteroides plebeius (AB200217); 5 = Bacteroides coprocola (AB200224); 6 = Bacteroides massiliensis (AY126616); 7 = Bacteroides gallinarum (AB253732); 8 = Paraprevotella xylaniphila (AB331897); 9 = Paraprevotella clara (AB331896); 10 = Prevotella copri (AB064923); 11 = Prevotella nanceiensis (AY957555); 12 = Barnesiella viscericola (AB267809); 13 = Barnesiella intestinihominis (AB370251); 14 = Parabacteroides merdae (AB238928); 15 = Paludibacter propionicigenes (AB078842); 16 = Pedobacter cryoconitis (AJ438170); 17 = Phascolarctobacterium faecium (X72865); 18 = Propionispira arboris (Y18190); 19 = Lutispora thermophila (AB186360); 20 = Gracilibacter thermotolerans (DQ117465); 21 = Natronovirga wadinatrunensis (EU338489); 22 = Caloramator australicus (EU409943); 23 = Clostridium clariflavum (AB186359); 24 = Acetivibrio cellulolyticus (L35516); 25 = Sporobacter termitidis (Z49863); 26 = Papillibacter cinnamivorans (AF167711); 27 = Eubacterium plautii (AY724678); 28 = Oscillibacter valericigenes (AB238598); 29 = Butyricicoccus pullicaecorum (EU410376); 30 = Clostridium sporospaeroides (X66002); 31 = Ruminococcus bromii (L76600); 32 = Ethanoligenens harbinense (AY295777); 33 = Clostridium cellulosi (L09177); 34 = Acetanaerobacterium elongatum (AY487928); 35 = Hydrogenoanaerobacterium saccharovorans (EU158190); 36 = Anaerotruncus colihominis (AJ315980); 37 = Ruminococcus albus (L76598); 38 = Ruminococcus flavefaciens (L76603); 39 = Desulfonispora thiosulfatigenes (Y18214); 40 = Desulfitobacterium metallireducens (AF297871); 41 = Desulfitobacterium chloroespirans (U68528); 42 = Desulfosporosinus auripigmenti (AJ493051); 43 = Clostridium irregulare ( [file 1757-4749-5-8-S5.tiff]
